# Weather, sex and body condition affect post-fledging migration behaviour of the greater flamingo *Phoenicopterus roseus*

**DOI:** 10.1186/s40462-023-00409-x

**Published:** 2023-08-23

**Authors:** Davide Scridel, Simone Pirrello, Simona Imperio, Jacopo G. Cecere, Giuseppe Albanese, Alessandro Andreotti, Giovanni Arveda, Fabrizio Borghesi, Giuseppe La Gioia, Luisanna Massa, Chiara Mengoni, Pierfrancesco Micheloni, Nadia Mucci, Riccardo Nardelli, Sergio Nissardi, Stefano Volponi, Carla Zucca, Lorenzo Serra

**Affiliations:** 1 Area Avifauna Migratrice (BIO-AVM), Istituto Superiore per la Protezione e la Ricerca Ambientale (ISPRA), via Ca’ Fornacetta 9, 40064 Ozzano dell’Emilia, BO Italy; 2 CNR-IRSA National Research Council-Water Research Institute, via del Mulino 19, 20861 Brugherio, MB Italy; 3 Department of Life Sciences, University of Trieste, 34127 Trieste, TS Italy; 4 Manfredonia, FG Italy; 5 Comacchio, FE Italy; 6 Servizio Tutela Ambiente e Territorio, Ufficio Zone Naturali, Comune di Ravenna, via Berlinguer 30, 48121 Ravenna, RA Italy; 7 Associazione Ornitologia Mediterranea, via Saponaro 7, 73100 Lecce, LE Italy; 8 Parco Naturale Regionale Molentargius Saline, via La Palma n 9, 09126 Cagliari, CA Italy; 9 Area per la Genetica della Conservazione (BIO-CGE), Istituto Superiore per la Protezione e la Ricerca. Ambientale (ISPRA), via Ca’ Fornacetta 9, 40064 Ozzano dell’Emilia, BO Italy; 10 Anthus s.n.c., via Luigi Canepa 22, 09129 Cagliari, CA Italy; 11 Area per i pareri tecnici e per le strategie di conservazione e gestione del patrimonio faunistico nazionale (BIO-CFN), Istituto Superiore per la Protezione e la Ricerca Ambientale (ISPRA), via Ca’ Fornacetta 9, 40064 Ozzano dell’Emilia, BO Italy

**Keywords:** Orientation and navigation, Partial migration, Migration phenology, Wetland, Movement ecology, Flight speed, Wind

## Abstract

**Background:**

Understanding which intrinsic and extrinsic factors dictate decision-making processes such as leaving the natal area or not (migratory vs resident strategy), departure time, and non-breeding destination are key-issues in movement ecology. This is particularly relevant for a partially migratory meta-population in which only some individuals migrate.

**Methods:**

We investigated these decision making-processes for 40 juvenile greater flamingos *Phoenicopterus roseus* fledged in three Mediterranean colonies and equipped with GPS-GSM devices.

**Results:**

Contrary to the body size and the dominance hypotheses, juveniles in better body condition were more likely to migrate than those in worse conditions, which opted for a residence strategy. Flamingo probability of departure was not associated with an increase in local wind intensity, but rather with the presence of tailwinds with departure limited to night-time mostly when the wind direction aligned with the migratory destination. Moreover, a positive interaction between tailwind speed and migration distance suggested that juveniles opted for stronger winds when initiating long-distance journeys. In contrast to previous studies, the prevailing seasonal winds were only partially aligned with the migratory destination, suggesting that other factors (e.g., adults experience in mix-aged flocks, availability of suitable foraging areas *en route*, density-dependence processes) may be responsible for the distribution observed at the end of the first migratory movement. We found potential evidence of sex-biased timing of migration with females departing on average 10 days later and flying *ca.* 10 km/h faster than males. Female flight speed, but not male one, was positively influenced by tailwinds, a pattern most likely explained by sexual differences in mechanical power requirements for flight (males being *ca.* 20% larger than females). Furthermore, juveniles considerably reduced their flight speeds after 400 km from departure, highlighting a physiological threshold, potentially linked to mortality risks when performing long-distance non-stop movements.

**Conclusion:**

These results suggest that not only intrinsic factors such as individual conditions and sex, but also extrinsic factors like weather, play critical roles in triggering migratory behaviour in a partially migratory metapopulation. Furthermore, social factors, including conspecific experience, should be taken into consideration when evaluating the adaptive processes underlying migration phenology, flight performance, and final destination selection.

**Supplementary Information:**

The online version contains supplementary material available at 10.1186/s40462-023-00409-x.

## Introduction

Animal migration is a widespread global adaptation that involves the movement of individuals in response to environmental conditions in order to improve their personal fitness (e.g. [12]). Despite the high energetic costs associated with migration, especially for species traveling long distances, the benefits typically outweigh the expenses compared to a year-round residency strategy [3, 16]. This is because exploiting the most seasonally suitable habitats at each stage in the life cycle can result in increased fitness for individuals, either through improved survival or breeding success [30, 63, 72].

Migration is most evident among birds, which have evolved a diversity of migration strategies varying greatly between species, populations, sexes, and age classes [53]. Strategies range from full migratory (all individuals display seasonal and directional movements) to partially migratory species (some populations/individuals migrate while others do not) or from short-distance and altitudinal migrants (species moving along an elevational gradient) to birds that undertake long-distance routes, crossing huge areas (i.e., long-distance migrants). Out of all, partial migration is arguably the most common form of migration [14], and likely represents a conditional strategy based on both intrinsic (e.g., genetics, physiology [47, 48]), and extrinsic (e.g., climate, resource availability, predation [26, 32]) factors. The individual choice of migrating or not in a partial migratory population is often influenced by behavioural differences, such as an individual’s response to adverse weather, competition for food, predator pressure, as well as intrinsic differences such as age and sex [35, 62]. However, the ongoing debate surrounding the factors that drive the decision to migrate or remain in place still requires further investigation, as scientists continue to call for more studies, particularly those employing biologging techniques, to provide a more comprehensive understanding of the mechanisms behind behavioral tactics within and among partial migrants [12, 35].

For individuals who migrate, migration phenology can also be affected by both endogenous (e.g., sex and age; [9, 54]), and environmental factors (e.g. [34]). In this regard, weather has also been shown to be an important trigger of bird migration, with various studies demonstrating the importance of tailwinds, weak or no crosswinds, low rainfall, high temperatures, and atmospheric pressure [21, 50, 65]. However, this might not always be the general rule as variations have been observed according to species, body size, and condition [53, 56, 73], as well as the geographical context [69]. In addition, as pointed out by Gallinat et al. [27], many studies have focused on such topics during pre-breeding migrations, whereas post-breeding and post-fledging migrations remain less investigated (but see [41, 43]).

With this study, we aim at assessing the role of specific intrinsic and extrinsic factors affecting migratory behaviour of juvenile flamingos belonging to a partial migratory population. We used bird-born GPS technology to investigate correlates for migration propensity and how weather influence migration departure, in-flight performance, and destination of juvenile birds. We focused on one of the most popular flagship species of wetland conservation, the greater flamingo *Phoenicopterus roseus* (hereinafter “flamingo”). This long-lived species, sexually dimorphic, with males being *ca.* 20% larger than females [38], has a complex pattern of movements in the western Palearctic, where it is described as migratory, partially migratory, dispersive, and at times erratic, [17, 19]. This complex spatial and temporal variation in flamingo movements is likely influenced by intraspecific competition, individual characteristics, social status, and stages of the reproductive cycle [18]. Winds may also play a fundamental role in determining the start and the migratory destination [22]. According to Green et al. [31], inter-annual cohort differences in the post-fledging distribution of flamingos ringed as chicks in France and spending the non-breeding period in Tunisia rather than in Spain, were associated with the annual variation in the prevalence of tailwinds assisting birds to Tunisia. Such findings suggest that wind plays a key role both in triggering the decision to start the migration and in determining the distribution of juveniles during their first non-breeding season. In this regard, the investigation of flamingo migration behaviour via modern biologging techniques may also provide novel information on flight performances (i.e., speed, duration, non-stop distance covered). Specifically, our research questions in this study were:

1. Can individual body condition predict the resident vs. migratory behaviour adopted within the context of the partial migration strategy of juvenile flamingos? We expect that birds in better body condition will be more likely to migrate than those in poorer condition, aligning with previous findings in this species [4, 67].
2. Does the migration phenology of juvenile flamingos vary between sexes? We do not expect to observe sex-biased migration patterns, such as protandry or protogyny, as these are typically observed in sexually mature or immature individuals that are prospecting for future nesting sites [43]. Furthermore, we anticipate that larger males will exhibit slower flight speeds compared to females due to the higher energetic demands associated with male flight, as described by Béchet [5].
3. Does the migration phenology of juvenile flamingos vary based on migration distance? According to La Sorte et al. [41], we expect that birds migrating longer distances will depart earlier than those covering shorter distances.
4. Do wind conditions at the colony site influence migration patterns? Based on the findings of Green et al. [31], we predict that short-term wind patterns will trigger the departure of birds from the colony site. Additionally, in line with Green et al. [31] research, we anticipate that migration destinations will align with prevailing seasonal winds at each colony. Moreover, we expect that migration distance is influenced by favorable winds, which facilitate long sea-crossings [44] and enable birds to reach a larger number of suitable wetlands.

## Materials and methods

### Study area

All flamingos tagged for the study were caught as chicks just before the fledging stage. The chicks were taken from three geographically distinct breeding colonies in Italy, each varying in both the year of establishment and colony size: i) Molentargius salt pans (Sardinia, 39°13′41.7″N 9°09′09.3″E) is the earliest Italian breeding site established in 1993 and currently hosts > 20,000 breeding pairs [55], ii) Comacchio lagoon and salt pans (Emilia-Romagna, 44°36′19.2″N 12°10′28.8″E), located near Po Delta, established in 2000 and currently the second largest colony in Italy, with > 7,400 pairs in 2022; and iii) Margherita di Savoia salt pans (Apulia, 41°23′55.4″N 16°02′25.5″E), established in 1996 and currently hosting *ca.* 2,000 breeding pairs. Margherita di Savoia is the only productive site for salt extraction while the other two salt pans are now non-productive and managed as bird conservation sites since three decades (i.e. before the establishment of their respective colonies).

### GPS deployment and sample analysis

We GPS-tagged 42 chicks across three years (2015, 2016 and 2017) during the first 10 days of August. Ringing activity followed a well-established protocol described by Johnson [37] with captures that took place when chicks were almost ready to fledge (i.e., when the oldest chicks of the colony were *ca.* 75 days old). Upon capture, tarsus length (to the nearest 1 mm), body mass (to the nearest 50 g) and wing length (to the nearest 1 mm) were recorded for each individual. Body feathers were sampled for molecular sex determination according to the following procedure. DNA was extracted from feathers calamus using the Qiagen® Dneasy Blood and Tissue Kit (Qiagen, UK) and the extraction process was performed using the automated QIAcube device (Qiagen, UK). Molecular sexing was carried out using a Polymerase Chain Reactions (PCR) following the protocol described in Mucci et al. [51]. Each individual was equipped with a solar powered GPS device with GSM transmission (Ecotone Telemetry, model SAKER-H and SAKER-L), using a Teflon harness. Devices were set to record GPS locations at 2 h intervals. Capture, handling and tagging procedures were carried out by the Italian Institute for Environmental Protection and Research (ISPRA), under the authorisation of Law 157/1992 [Art. 4(1) and Art. 7(5)], which regulates research on wild bird species in Italy. Harness and GPS weight (range 19.4–25.9 g) amounted to far less than 3% of body mass (0.3–0.9%) in all individuals [6].

### Defining juvenile migration departure and arrival

We considered a post-fledging migratory movement when a juvenile flamingo departed for a flight beyond a 100-km radius from the natal colony and arrived at the non-breeding site before the 1st of November of the hatching year. Visual inspection of initial movements in GIS environment (“QGIS”, [60]) confirmed that 100 km was an appropriate cut-off, and that arrival at non-breeding sites was indicated by a transition from directed, long-distance flights to a series of smaller-scale movements. Ultimately, we relied on GPS data of between 26 and 32 migrating flamingos (2 GPS devices stopped working immediately after tagging while 8 juveniles did not migrate) depending on the analyses performed (see Results and Additional file 1: Tables S1, S2). For each migratory individual, departure was defined as the first GPS location on a flight after leaving the colony on migration. For the purposes of the present work, the end of the migratory movement was defined as the arrival at the first location after a straight, uninterrupted flight of more than 100/km day directed to a wetland where flamingos stayed either (i) for at least 12 h (i.e., stop-over site) or (ii) for a minimum of 20 days (non-breeding site). For each individual the migration track was therefore composed of a departure location (natal colony), intermediate in-flight locations, and an arrival location.

### Weather data

We used the environmental-data automated track annotation (Env-DATA) system [20] to associate the tracking data with atmospheric observations obtained from ECMWF Global Atmospheric Reanalysis models. Weather conditions included: temperature (“ERA5 PL Temperature”), precipitation (“ERA5 Total Precipitation”), relative humidity (“ERA5 Relative Humidity”), atmospheric pressure (“ERA5 Surface Air Pressure”), wind as U (zonal or west/east) and V (meridional or south/north,“ECMWF ERA5 PL U/V Wind”) flow components all at 0.25° resolution and 1 h temporal granularity. Cloud cover was instead derived from Interim data for low, medium and high altitude with a spatial granularity of 0.75° and a temporal granularity every 6 h. All weather data was interpolated bilinearly to match GPS locations spatially and temporally as per Dodge et al. [20]. For wind we derived its direction and intensity using the “*rWind*” package [23]. To calculate the tailwind component, we used the formula *v* × cos* x*; where *v* is the wind speed in ms^−1^ and *x* is the angular deviation between the flight direction (i.e., headwind/tailwind direction) and the wind direction (in degrees). For crosswinds, we used the formula *v* × sin* x* and its absolute value, which represented the strength of the wind (in km/h) component blowing perpendicular to a given movement track, regardless of the direction from which it originates. Tailwind values (in km/h) ranged from negative to positive, where negative values indicated headwinds and positive values indicated tailwinds. The calculation of tailwind and crosswind components was dependent on the type of analysis being conducted. When assessing short-term (3 days) weather correlates on the probability of departure (Section Short-term environmental correlates triggering migratory departure, Table 1) and the impact of prevailing seasonal winds on migration direction (Section Seasonal correlates of migration direction, Additional file 1: Table S8), the flight directions were determined by calculating the angle between the departure and arrival location. On the other hand, when evaluating speed, flight directions were based on the angle between consecutive locations during the flight (Section Migration speed, Table 2).

**Table 1:** Binomial GLMM summary describing the probability of migration departure with respect to weather conditions experienced during the 3 days preceding departure and the departure day

Probability of migration departure					
Predictors	Estimate [95% CI]	SE	χ^2^	df	*P*
Intercept	− 4.97 [− 7.49, − 2.44]	1.69	14.92	1	0.001
Crosswind	− 0.27 [− 1.08, 0.53]	0.41	0.45	1	0.503
Wind speed	0.68 [− 018, 1.55]	0.44	2.38	1	0.120
**Tailwind**	**1.41 [0.75, 2.07]**	**0.34**	**17.57**	**1**	** < 0.0001**
Air pressure	0.5 [− 0.54, 1.55]	0.53	0.89	1	0.345
Humidity	− 0.51 [− 1.30, 0.28]	0.4	1.6	1	0.205
Cloud cover (medium)	− 0.53 [− 1.85, 0.80]	0.68	0.6	1	0.438
Cloud cover (high)	− 0.11 [− 0.57, 0.35]	0.23	0.22	1	0.634
Cloud cover (low)	− 0.4 [− 1.64, 0.83]	0.63	0.41	1	0.523
Temperature	0.96 [− 0.28, 2.19]	0.63	2.31	1	0.128
Rain	0.19 [− 0.42, 0.79]	0.31	0.36	1	0.548
**Colony (Margherita di Savoia)**	**3.09 [0.28, 5.91]**	**1.43**	**7.03**	**2**	**0.031**
Colony (Molentargius)	0.57 [− 0.28, 3.42]	1.45	7.03	2	0.695
Year (2016)	− 0.14 [− 2.24, 1.97]	1.07	0.017	2	0.898
Year (2017)	− 0.17[− 3.40, 3.07]	1.65	0.017	2	0.919
Sex (Male)	− 0.36 [− 1.54, 0.83]	0.6	0.347	1	0.555
**Hours before sunset**	**4.37 [2.34, 6.41]**	**1.04**	**17.78**	**1**	** < 0.0001**
**Hours before sunset**^**2**^	** − 2.52 [− 3.89, − 1.14]**	**0.7**	**12.87**	**1**	**0.0003**

Response variable was coded as “0” (n = 676) representing all locations 3 days prior to departure and as “1” (n = 30) the first location in flight. Predictors included sex, colony (reference level: Comacchio), year (reference level: 2015), and weather variables. Standardized estimates are shown alongside 95% confidence intervals, test statistics (Wald’s χ^2^, type *III*), degrees of freedom (df) and *p*-value (*P*). Predictors with significant *p*-values (α = 0.05) and with 95% confidence intervals not overlapping zero are shown in bold. Effects of biometrics and their interaction with weather variables were assessed preliminary and excluded as they were considered to have little influence

**Table 2:** Summary of LMM estimating correlates of variation in consecutive speed during flamingos’ post-fledging migration

Migration speed					
Predictors	Estimate [95% CI]	SE	F	df	*P*
Intercept	72.05 [62.15, 81.96]	5.05	13.19	1	< 0.0001
Year (2016)	− 1.38 [− 19.33, 16.56]	9.15	25.58	1	0.880
**Sex**	** − 10.73 [− 18.30, − 3.17]**	**3.86**	**18.86**	**1**	**0.013**
Crosswind	− 2.56 [− 5.71, 0.60]	1.61	110.02	1	0.121
**Tailwind**	**8.52 [5.53, 11.52]**	**1.52**	**65.77**	**1**	** < 0.0001**
Cloud cover (high)	− 1.04 [− 4.05, 1.98]	1.54	48.63	1	0.519
Cloud cover (low)	− 1.06 [− 5.20, 3.07]	2.12	111.73	1	0.620
Cloud cover (medium)	− 1.78 [− 5.74, 2.18]	2.02	49.08	1	0.396
Temperature	5.05 [− 2.09, 12.21]	3.64	67.82	1	0.185
**Humidity**	**0.22 [− 4.05, 4.51]**	**2.18**	**71.12**	**1**	**0.921**
**Humidity**^**2**^	** − 4.17 [− 6.35, − 2.0]**	**1.11**	**112.73**	**1**	** < 0.0001**
Rain	0.96 [− 2.57, 4.51]	1.81	106.93	1	0.596
Air pressure	− 0.54 [− 4.30, 3.21]	1.91	112.85	1	0.779
Colony (Margherita di Savoia)	− 5.96 [− 26.47, 14.55]	10.46	23.72	2	0.574
Colony (Molentargius)	− 8.15 [− 30.36, 14.05]	11.33	23.72	**2**	0.476
**Cumulative distance**	**8.2 [4.43, 11.97]**	**1.92**	**112.98**	**1**	** < 0.0001**
**Cumulative distance**^**2**^	** − 8.79 [− 11.41, − 6.18]**	**1.33**	**113.00**	**1**	** < 0.0001**

Predictors included sex, year (reference level: 2015) breeding colony (reference level: Comacchio), weather variables, and cumulative distance (i.e. the total distance travelled up to the position where the consecutive speed was evaluated). Standardized estimates are shown alongside 95% confidence intervals, standard error, test statistics (Kenward-Roger approximation F test), degrees of freedom (df) and *p*-value (*P*). Predictors with significant *P*-values (α = 0.05) and with 95% confidence intervals non overlapping zero are shown in bold

### Statistical analysis

Prior to assessing morphological, body condition, and weather drivers of migration decision, phenology, probability of departure, speed, and distance (see below for details), we performed a priori analysis to assess whether morphological and body condition, varied depending on sex, natal colony, and year. Gaussian distributed linear models (LMs) were run to assess potential differences for each biometric value (response variables: tarsus length, body mass, wing length) in relation to factors such as sex, natal colony (including an interaction term between the latter two) and year. For an index of body condition, we calculated the Scaled Mass Index (“SMI”; [58]) separately for males and females. Male tarsus was positively correlated with body mass (r = 0.60, n = 31, *P* = 0.001) but the relationship was less clear for females possibly due to the lower sample size (r = 0.27, n = 11, *P* = 0.27). SMI for each individual *i* was still computed as: *SMI*_*i*_ = *body mass*_*i*_ × *(tarsus*_*0*_*/tarsus*_*i*_*)*, where tarsus_0_ for males was 275 mm and for females 247 mm (mean tarsus of the sampled population).

To test the prediction that birds in better body condition migrated with higher probability than birds in poorer body condition [4], we created a dichotomous variable coding as “0” juveniles that did not migrate (n = 8) and with “1” birds that migrated away from the natal colony before the 1st of November in the respective year (n = 32). This was used as a response variable in a logistic regression model in order to assess the probability of migration in relation to SMI (explanatory variable).

For migrated individuals, we assessed drivers of migration phenology (i.e. departure date) by fitting a series of LMs setting migration day (expressed as day of year, 1 = 1st of January) as a response variable with normal distribution as a function of sex, colony (including an interaction term between the latter two), tagging year, and total migration distance as explanatory variables. Morphometric variables (i.e., body mass, tarsus, and wing length) and SMI were also added as explanatory variables in the models, but were tested individually to avoid collinearity and overfitting.

To model the probability of (migration) departure as a function of weather variables we used a generalised linear mixed model (GLMM) with binomial error distribution and a logit-link function. The response variable was coded as “0” for all locations between 04:00 pm and 02:00 am (local time) within 3 days prior to departure, as according to our data and existing literature [36], flamingos mostly departed at night (mean = 09:20 pm, range: 06:00 pm–02:00 am, local time, own data). For each individual, we allocated the value “1” for the first location in flight towards the migratory destination but we attributed to this the weather conditions at the colony. In this way, we provided information to the model on the conditions that triggered birds’ departure from the colony rather than during flight. In addition to weather variables, in the model we included other fixed factors, namely colony, year, and hour of migration (expressed as hours after sunset), while we set bird identity as a random effect to account for repeated locations of the same individuals. We included hours after sunset in the model, along with weather variables, to disentangle effects between a visual trigger (decrease in light at dusk) and other variables (such as tailwinds, temperature, and humidity) that could peak at certain times of the day (i.e., sunset). We opted to exclude biometrics and SMI to avoid inflating the number of variables, considering that no effect was detected in the previous analysis dealing with correlates of migration departure date (see Results section “Migration probability and phenology”). Given the significance of tailwinds for long-distance migrations, we tested the hypothesis that birds traveling greater distances exhibit a preference for stronger tailwinds. To investigate this, we fitted a GLMM with the same fixed and random structure described above, but included an assessment of the interaction between migration distance and tailwind (fixed effects).

To examine the potential impact of weather variables, sex, and biometrics on the migration speed of the flamingos, we computed relative speeds based on the distance and time information between consecutive flight locations. We excluded speed data pertaining to the final segment of the migration track to prevent the detection of decelerating effects attributed to the birds’ arrival, and also because we lacked precise information on the exact moment of arrival due to the coarse GPS locations being recorded at 2-h intervals. We used linear mixed models (LMMs) to model the consecutive speed values between flight locations (response variable Gaussian distributed) as a function of weather variables (linear and quadratic terms) while accounting for sex, colony, year, and cumulative distance migrated since leaving the colony (i.e. the total distance travelled up to the position where the consecutive speed was evaluated) to evaluate potential effects of exhaustion (decreased speeds) over long distances. Bird identity was set as a random effect to account for repeated measurements of speed along the migration route. As preliminary tests suggested no effects of biometrics including potential interaction with any of the weather variables used, we opted to exclude them from the model.

**Fig. 1:**
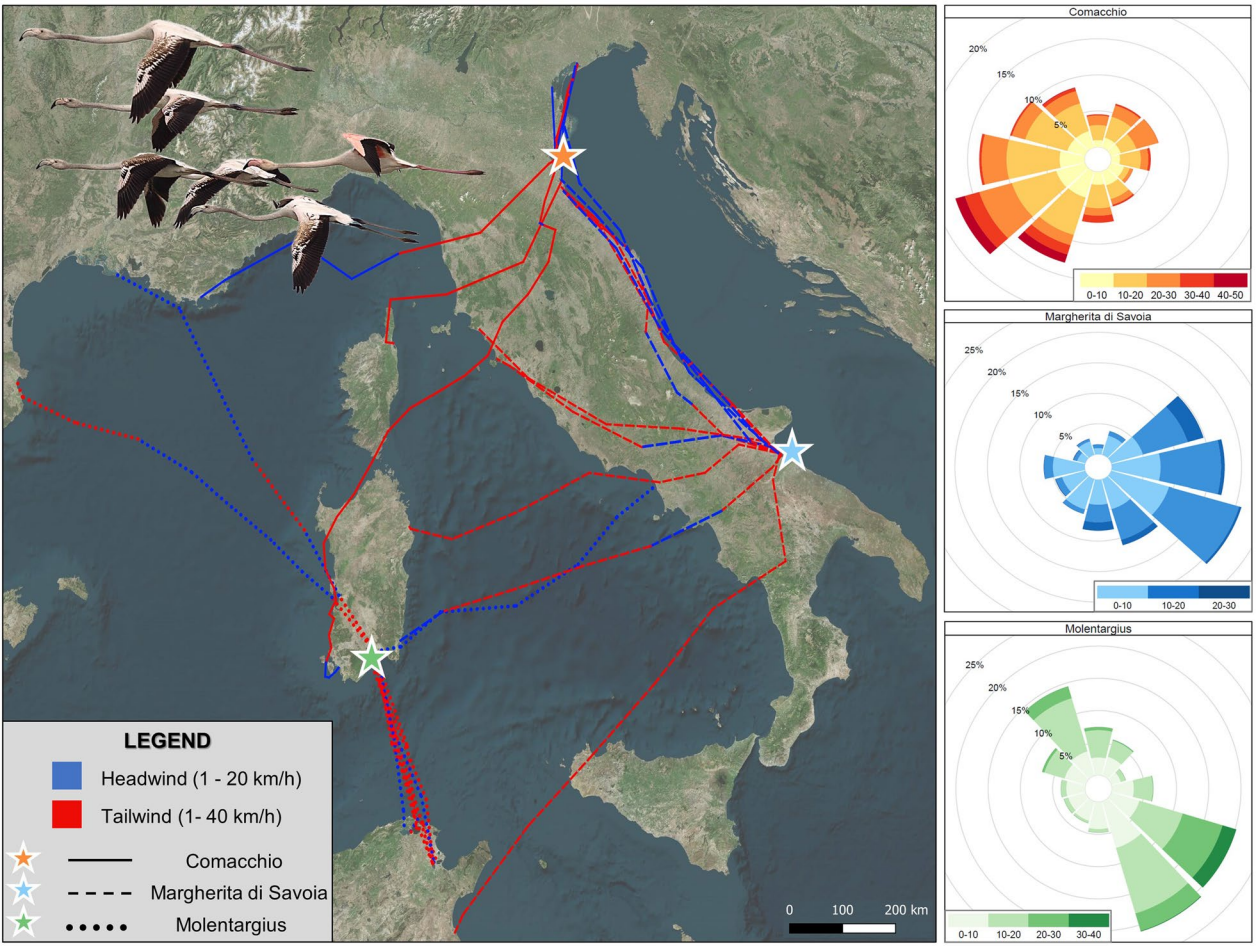
Post-fledging migration tracks of 32 juvenile greater flamingos from three different colonies located in Italy. The locations of the natal colonies (Comacchio, Margherita di Savoia and Molentargius) are represented by different-coloured stars, while migration routes are represented with solid, dashed and dotted lines according to the natal colony of each individual (see legend). Headwinds and tailwinds experienced during migration are represented in blue and red respectively. The panels on the right show the prevailing wind conditions experienced by each colony from the day of the first flamingo departure to the last. The rose charts show the blowing wind direction (not the wind origin) with the horizontal bars representing the speed (km/h)

To test the prediction that prevailing seasonal winds influenced the migratory direction (and consequently the final destination), we used the destination direction (i.e. angular direction between last location in the colony and the arrival location) of each bird at each colony as a proxy of migration route. This was a reasonable choice, as we had no evidence of strong deviations of migratory routes from departure to arrival: once a juvenile departed from the colony, it did not strongly deviate from its initial direction (see linearity of routes in Fig. 1). Seasonal winds were then calculated using the frequency of wind directions for the entire migration period (from the day of the first flamingo departure to the last) at each colony. However, since both wind data and departure directions were not always unimodal and in some cases they were clearly bimodal (Railegh test for wind direction in Molentargius: t = 1, *P* = 0.51; Comacchio: t = 0.37, *P* < 0.0001; Margherita di Savoia: t = 0.41, *P* < 0.0001; Railegh test for bird departure direction in Molentargius: t = 0.64, *P* = 0.003; Comacchio: t = 0.31, *P* = 0.47; Margherita di Savoia: t = 0.49, *P* = 0.003; tests performed via “*circular*” package [1]), we decided not to use a circular test to verify the relationship between migratory and seasonal winds directions, but to categorise data by grouping the angular direction of winds into 6 classes each of them representing a span of 60° (i.e., 0°–60°, 61°–120°, 121°–180°, 181°–240°, 241°–320°, 321°–360°). In this way we were able to meet linear model assumptions, and the frequencies of angular departure of birds in the 6 classes at each colony (response variable) were then correlated via GLMs with a Poisson error structure with the wind directional frequencies. Models were checked for issues associated with overdispersion and zero-inflation via the R package “*DHARMa”* [33], but these were not detected.

Weather correlates of migration distance were assessed via LMs with the response variable being the sum of the Euclidean distances between consecutive GPS locations from departure (last location in the colony) to arrival location (first location at arrival destination) which were calculated using the “*geodist*” package [57]. LMs were used to model the total distance performed by each flamingo (response variable Gaussian distributed) as a function of weather variables averaged across the migration route while accounting for sex, colony, and migration date by including them as explanatory variables.

All analyses were performed in R software (version 4.0.4) and the models were tested for within-group collinearity by calculating the variance inflation factor (VIF) using the package ‘*car*’ [25]. Explanatory variables with VIF value ≥ 3 [76] were removed from the model and multicollinearity was re-checked to verify that the remaining variables were not correlated. Variables were scaled and centred at the mean for better interpretation of coefficient and to improve model convergence.

## Results

### Differences in morphology and body condition

When assessing potential morphological and body condition differences across all chicks captured for tagging (n = 42) in relation to sex, natal colonies and years, we found that the chicks were on average larger (i.e. body mass, tarsus and wing length) in Comacchio compared to Margherita di Savoia and Molentargius, suggesting an earlier hatching of chicks captured in Comacchio. This seems evident despite not knowing exact laying or hatching dates. Chicks caught in 2015 were also smaller than those captured in 2016 and 2017. Chicks in Margherita di Savoia had a larger SMI when compared to Comacchio and Molentargius (Additional file 1: Table S3). Tarsi were significantly longer in males than in females whereas no sex differences in wing length or body mass were detected. There was some limited evidence (*P* = 0.07) that males were in better body condition, recording higher SMI values than females (Additional file 1: Table S3, Figure S1–S4).

### Migration probability and phenology

**Fig. 2:**
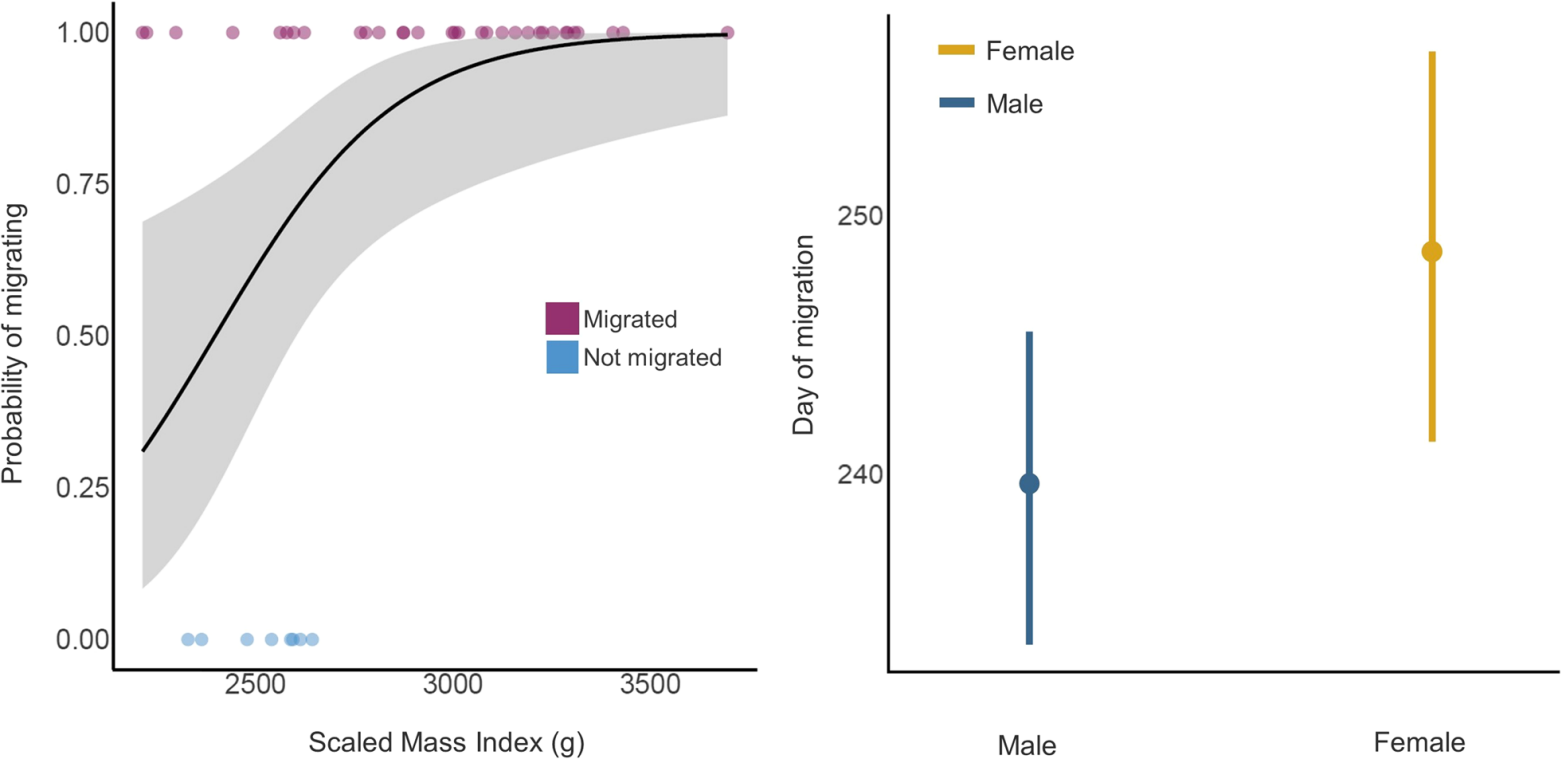
Left panel: Probability of migrating in relation to Scaled Mass Index (SMI) according to the GLMM reported in Additional file 1: Table S4. Purple dots represent raw data of departing individuals (coded as “1”; n = 32) while not migrating individuals are shown in blue (coded as “0”; n = 8). Right panel: sex difference in migration departure date (day of year) when accounting for SMI, colony and year, according to the linear model reported in Additional file 1: Table S5

Birds in better body condition at the time of tagging had higher probability of migrating (n = 32) whereas birds with a lower SMI showed a preference for not migrating (*P* = 0.001, n = 8; Additional file 1: Table S4, Fig. 2). Overall, the amount of variance explained by a model including SMI was moderately high (McFadden’s R^2^ = 0.33; Additional file 1: Table S4). High percentages of both sexes migrated and there was no difference in the likelihood of migrating in relation to sex (migrating females 10/11—90.91%; migrating males 22/29–75.86%; X^2^ = 0.05, df = 1, *P* = 0.81).

Migration departure dates ranged between the 6th of August and the 16th of October and varied across individuals, colonies, and year (Additional file 1: Table S2). Body mass, tarsus, wing, SMI, and migration distance did not predict migration timing (i.e., the flamingos’ departure date), which was instead strongly predicted by colony across all models (adj-R^2^ = 0.73–0.80; Additional file 1: Table S5). Indeed, birds from Margherita di Savoia departed *ca.* 20 days later than those from Comacchio and *ca.* 35 days later from those of Molentargius (Additional file 1: Figure S5). Models accounting for SMI as predictors showed evidence (*P* = 0.03) for an earlier departure by males of *ca.* 10 days (Fig. 2, Additional file 1: Table S5).

### Short-term environmental correlates triggering migratory departure

**Fig. 3:**
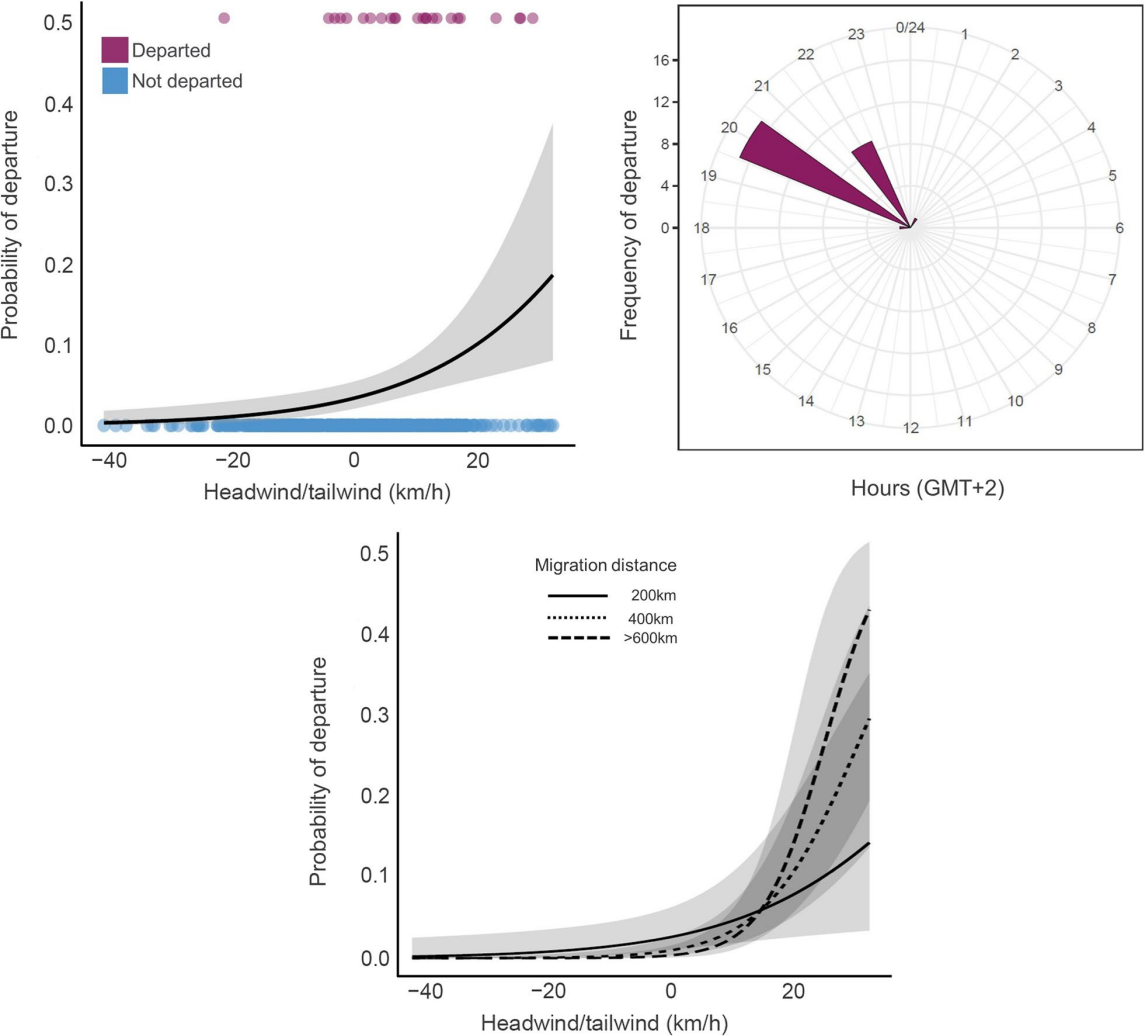
Fitted relationships (Table 1) for the most meaningful variables (top left: tailwind; top right: time of departure) describing the probability of departure of juvenile greater flamingos (n = 30). The bottom panel shows the probability of departure in relation to headwind/tailwind speed according to migration distance: flamingos migrating longer distances had a higher probability of selecting stronger tailwinds at departure than those migrating shorter distances (Additional file 1: Table S6). In the top left plot, the purple dots represent the raw data of headwind/tailwind condition experienced during migration departure, while the blue dots represent conditions three days prior to the departure

For migratory flamingos, the probability of departure was higher when they experienced greater tailwind (µ = 8 km/h, SE = 0.67, range = −9.48, 29.94; Table 1, Fig. 3) compared to the 3 days preceding the departure from colony. Flamingos migrated mostly at night, with the highest probability of departure being just after sunset. Fixed effects accounted for a good proportion of the variance in the model (marginal R^2^ = 0.45; [74] with time of departure being the most important predictor (semi-partial R^2^ = 0.36), followed by tailwind (semi-partial R^2^ = 0.09). We found no support for effects of wind intensity, crosswind, atmospheric pressure, temperature, cloud cover, humidity, and precipitation nor for interacting effects of weather variables with either sex or colony (results assessed in preliminary analyses). A model accounting for the interactive effects of tailwind and migration distance indicated that birds traveling longer distances selected significantly stronger tailwinds than those migrating shorter distances (*P* = 0.02; Additional file 1: Table S6; Fig. 3).

### Migration speed

**Fig. 4:**
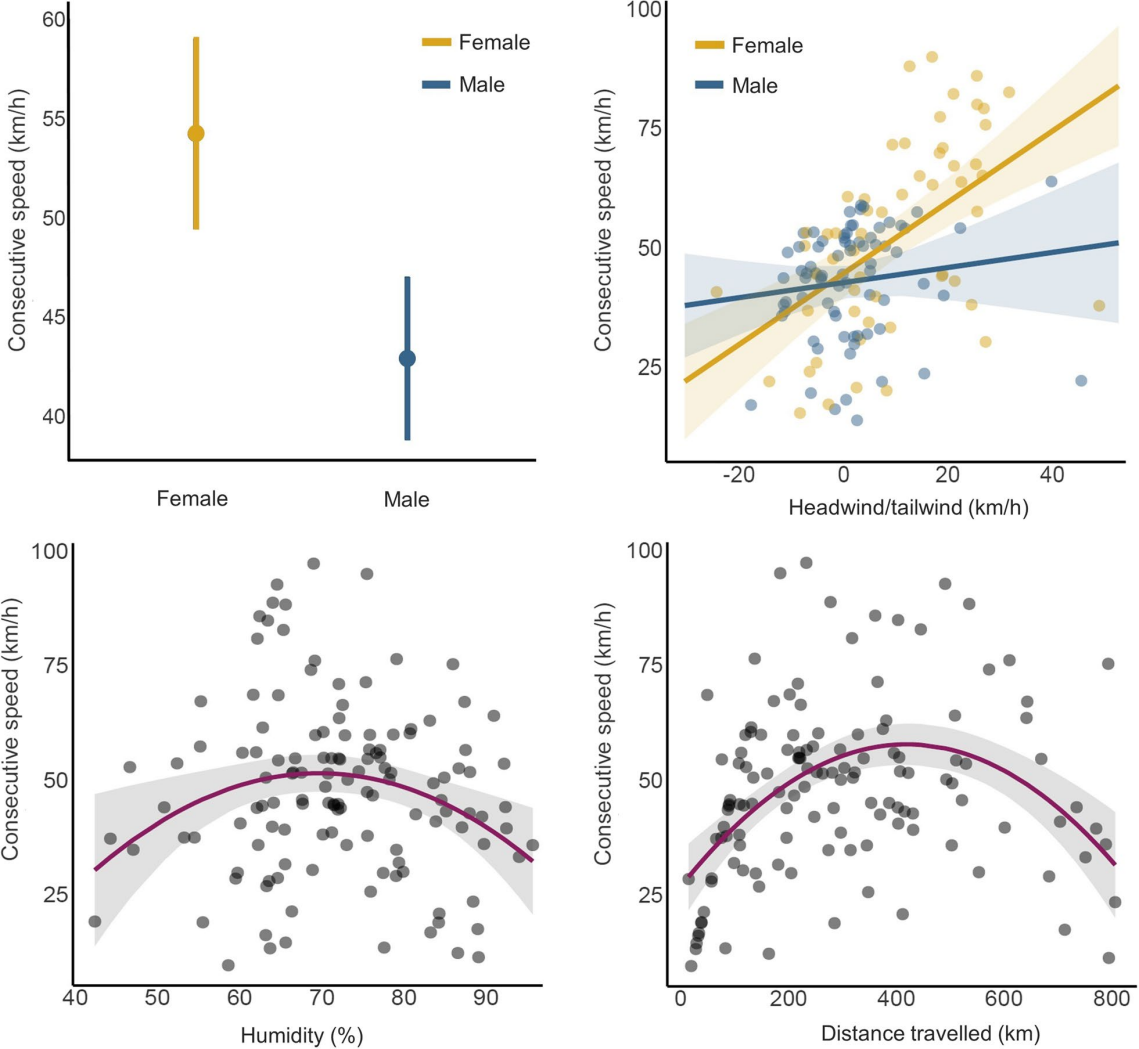
Relationships for the most important variables associated with variations in migratory speed across consecutive locations for juvenile greater flamingos, according to the model reported in Table 2 and Additional file 1: Table S7 for the interaction term between sex and tailwind (plot on the top right)

The mean migratory speed recorded by flamingos was 54.6 km/h (range = 9.5, 97.2, n = 26) (Additional file 1: Table S2). Based on the data from our models (Table 2), we observed a significant difference in migration speed between males and females. Specifically, females had an average flying speed of 10 km/h faster than males (Fig. 4). A model strictly focused on the interaction between sex and tailwind condition suggested that female speed was more influenced by tailwinds than males (*P* = 0.009, Additional file 1: Table S7, Fig. 4). Migration speeds were also influenced quadratically by humidity (peaking around 70%) and distance travelled, with birds reducing their speed after journeys of *ca.* 400 km. The model explained a good proportion of the variance (marginal R^2^ = 0.54; [74]), with total distance being the most important predictor (semi-partial R^2^ = 0.15) followed by tailwind (semi-partial R^2^ = 0.11) and sex (semi-partial R^2^ = 0.05).

### Seasonal correlates of migration direction

**Fig. 5:**
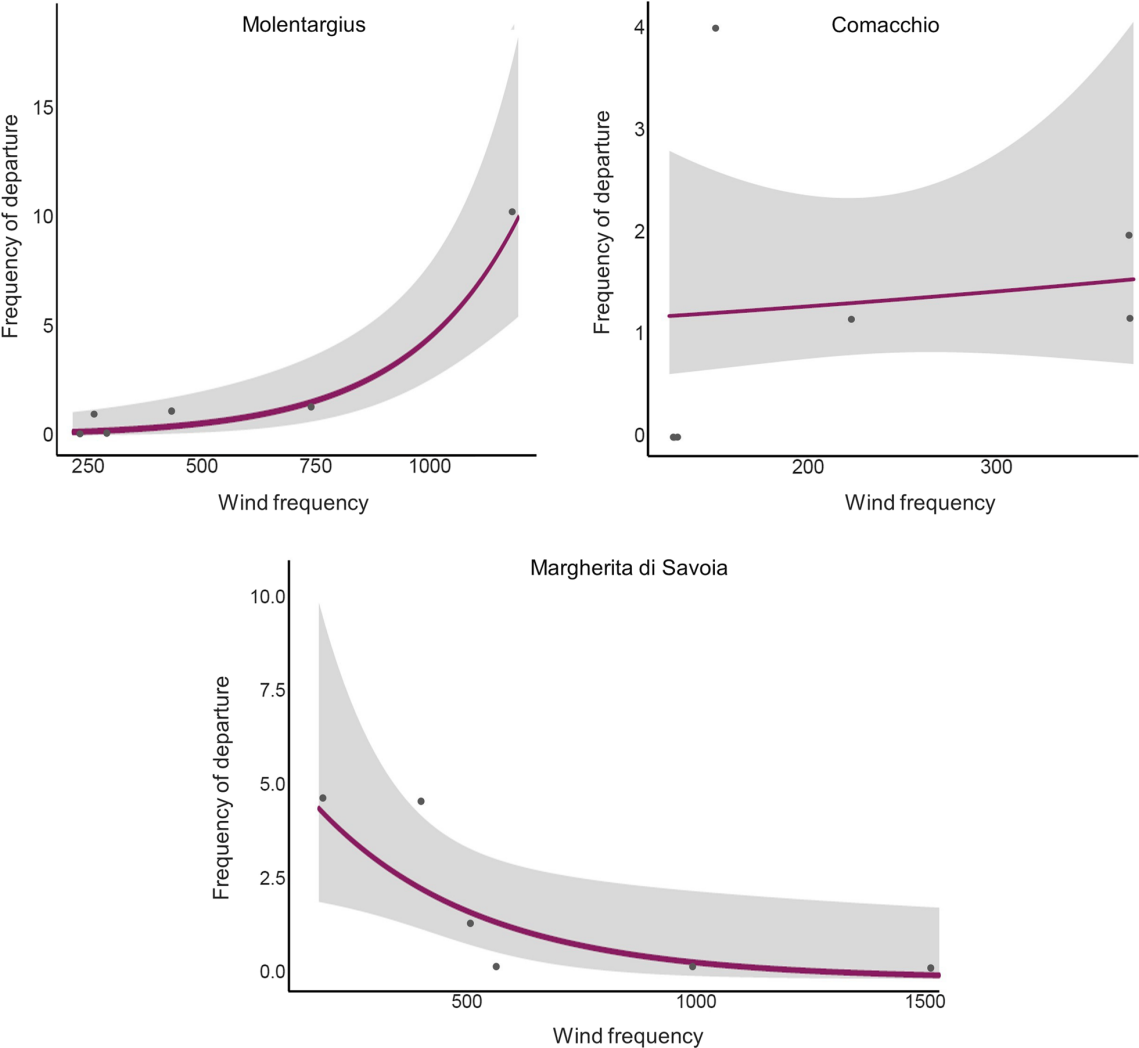
Linear relationship between the frequency of departures in given migration directions and the seasonal frequencies of winds. The data, which is presented in Additional file 1: Table S8, includes the number of flamingos that departed toward a certain angular direction slot (n = 32) and the number of times (at two-hour intervals) that wind blew at a certain angular direction for each colony over the season. Both bird and wind directions were categorised based on 6 direction classes, each consisting of 60°, and the frequency of seasonal winds were calculated from the day of the first flamingo departure to the last one for each colony. A positive relationship between bird departures and wind frequency suggests a potential influence of seasonal winds on birds’ destination

During the post-fledging migration, the 32 migrating individuals covered on average 388 km (range: 103–813 km), reaching sites located in Italy, France, Spain, and Tunisia (Fig. 1). Most juveniles (i.e., 76.9%; n = 10/13) born in Molentargius migrated to Tunisia, while 7.7% (n = 1/13) went to Spain, 7.7% (n = 1/13) to France (i.e., Camargue), and 7.7% (n = 1/13) to Margherita di Savoia. Five out of 8 (i.e., 62.5%) juvenile flamingos from Comacchio colony migrated to the nearby Lagoon of Venice, 12.5% (n = 1/8) headed to Sardinia, 12.5% (n = 1/8) to Corsica, and 12.5% (n = 1/8) to France. Flamingos born in Margherita di Savoia showed heterogeneous migration destinations: 45.4% (n = 5/11) of juveniles flew northwards towards the Po Delta, 18.2% (n = 2/11) to Sardinia, 18.2% (n = 2/11) to Tuscany, 9.1% (n = 1/11) to Tunisia, and 9.1% (n = 1/11) to the Lagoon of Venice. Only for Molentargius, the frequency of departures in particular migration directions (angle between the last location in the colony and arrival destination), categorised in 6 classes, was positively correlated with the frequencies of seasonal wind directions experienced at the colony (*P* < 0.0001) whereas these were negatively correlated for Margherita di Savoia (*P* = 0.01) and no relationship was found for Comacchio (*P* = 0.68; Figs. 1 and 5, Additional file 1: Table S8).

### Migration distance

Colony and year were the most important variables in predicting migration distance (Additional file 1: Table S9, Figure S6), accounting for 63% of the variation of the model. Flamingos from Margherita di Savoia and Molentargius flew two to three times greater distances than those from Comacchio (Additional file 1: Figure S6). We found no influence of sex and weather variables on the migration distance performed by juvenile flamingos. Potential effect of body condition was assessed a priori before fitting all weather variables and excluded from the modelling approach as found to be not significant (Scaled Mass Index, *P* = 0.52).

## Discussion

Assessing intrinsic and extrinsic factors shaping the migratory behaviour of a partial migrant species has implications for understanding how migration processes take place. GPS data analysis from three Mediterranean colonies of flamingos revealed that the decision to undertake the post-fledging migration depended on physical condition, with juveniles in better condition being more likely to migrate compared to those in worse condition, which remained near their natal colony. All migratory individuals departed just after the local sunset, taking advantage of more favourable tailwind conditions compared to 3 days prior to departure, with stronger tailwinds being selected when birds had to travel longer distances. Despite the limited sample size, we still detected sex-related differences in the timing of migratory departure and speed, with males, which are larger than females, departing earlier in the season and performing their migratory flight at a slower speed. Average migration speed peaked at around 400 km distance from the natal colony, after which it declined drastically, suggesting potential energetic constraints for juvenile birds migrating long distances. Migratory directions were influenced by the direction of prevailing seasonal winds only for one population.

### Body condition influencing migration behaviour

A key finding in our study was that the decision to migrate (80% of tagged juveniles) or stay in the natal area (20% of tagged juveniles) most likely depended on body condition during the final stages of growth. Despite the smaller sample size, our findings mirror Barbraud et al. [4] who used re-sightings of ringed individuals to show that the probability of movement of juvenile flamingos from the natal colonies in the Camargue (southern France) to their main non-breeding grounds (Spain, Sardinia, Tunisia, and France) was higher for individuals in good body condition. Such a result contrasts with the body size and dominance hypotheses of bird migration which suggest that larger individuals and those in better body condition are likely to remain in breeding or natal area since they can successfully outcompete subordinate animals for food and endure cold-related stress more successfully [7, 28]. We argue that the propensity of flamingos to migrate despite or due to their body condition could be explained by various hypotheses, in part already discussed in previous studies (e.g., [4]). Birds may disperse from natal areas to spend the non-breeding period on safer grounds, thus avoiding unfavourable cold weather that is known, in this species, to cause high mortality rates and feeding difficulties, such as frozen wetland [42]. In this scenario, only birds in good condition are able to reach distant non-breeding grounds without increasing the mortality risk. While we believe that food might be a limiting factor for this species, it is unlikely to be the only trigger for migration. As pointed out by Barbraud et al. [4] for Camargue, also Molentargius, Margherita di Savoia and Comacchio annually hold large numbers of flamingos during the non-breeding season, suggesting that the wetlands hosting the colonies are not food-depleted at the end of breeding season, but only a low proportion of this total are juveniles (authors’ pers. obs). Intraspecific competition, driven by age-related behavioural dominance and density-dependence processes, might also be the cause of migration. For example, juvenile American flamingos *Phoenicopterus ruber ruber* were frequently recipients of aggression during foraging by adults, which decreased the amount of time available for their own foraging [70]. Despite the limited sample size, our models suggested that females and males had a similar probability of migrating (females: 90.9%; males: 75.9%). This result is also confirmed by previous studies assessing sex-related differences in post-fledging and post-breeding flamingos which used individual re-sightings of colour-marked birds. Despite their larger sample sizes, they did not detect any difference between the sexes [4, 52].

Colony identity was found to be the primary predictor of migration phenology. Juveniles from Margherita di Savoia exhibited a delay of approximately 20 days compared to those from Comacchio, and a delay of about 35 days compared to those from Molentargius. However, understanding the underlying reasons for these differences is challenging due to the involvement of multiple factors. Potential explanations could include variations in intercolonial laying and hatching dates, fluctuations in water level regimes due to hydrological management practices in wetlands and salt pans (which were not known in this study), and density-dependent effects such as competition for food. Interestingly, we found some limited indications that female flamingos delayed post-fledging migration by *ca.* 10 days with respect to males. Sex-biased timing of migration has most often been demonstrated for arrival on the breeding grounds and less for non-breeding areas [49]. Briedis et al. [9] analysed 350 migratory tracks belonging to 15 passerine species and found that males generally depart earlier than females from the breeding sites. Earlier departures of males are generally attributed to their shorter investment in reproduction and to the shorter distance they cover to reach the non-breeding areas [8]. As our analysis focused on juvenile birds (sexual maturity is reached at 3 years old [38]), the above-mentioned explanations have little relevance to our findings unless we hypothesize that both flamingos, adults and juveniles, migrate in partially sexually segregated flocks. We approach these results with caution as further studies are required to assess the mechanisms underlying such behaviour using a larger sample size.

### Tailwinds influence migration departure and speed, albeit to a limited extent the direction

In our work, 73% (n = 22/30) of birds departed with tailwind conditions, while wind speed alone was not a supported variable in the model suggesting that birds waited for a specific wind direction towards the potential destination, rather than strong wind conditions, before taking off. The average migratory speed recorded by flamingos was 45.2 SE ± 1.6 km/h, reaching a peak of 97.2 km/h with tailwind assistance, while the longest journey recorded was a 18 h uninterrupted flight. Such results seem in line with previous findings (see [66] and references therein), but our work is the first to have detected sex differences in flying speed, with males flying on average slower than females by *ca.* 10 km/h (Fig. 4). Female flight speed appeared to be positively influenced by tailwinds, while males flew at a more or less constant speed notwithstanding variations in headwind/tailwind conditions. Sexual differences in flight performance may be attributed to differences in mechanical power requirements for flight. Indeed, according to Béchet [5], the power required for adult males is almost twice that required for females. Although our biometric analysis during tagging did not reveal any obvious differences in morphology, except for males having longer tarsi, we speculate that sexual dimorphism may have developed as the fledglings matured, becoming more pronounced at the time of migration. This could explain the observed differences in migratory speeds between the sexes.

By comparing the distribution of seasonal winds at each colony during the migration period with the direction of the first migratory flight, it was possible to test if destinations aligned with prevalent winds (Fig. 1). According to our analysis, this relationship occurred only for Molentargius birds, but not for birds from Comacchio and Margherita di Savoia (Fig. 5). The Mistral, a strong wind blowing from southern France towards the Mediterranean, had clear influence on the migration destination of birds born in Molentargius, with *ca.* 77% of individuals flying southwards towards Tunisia. Such finding mirrors Green et al. [31] observations in the Camargue (France), which hosts a large flamingo colony and where the strongest Mistral winds occur. Here, the birds take advantage of Mistral to increase their flight speed by *ca.* 30% and perform long sea-crossings to reach Sardinia and Tunisia [44]. This is also confirmed by the significant interaction detected between tailwind and migration distance suggesting that birds were more likely to choose a stronger tailwind during departure when they needed to cross longer distances, possibly indicating the determinant role of experienced adult birds in mixed-age flocks in establishing migratory destinations (Additional file 1: Table S6, Fig. 3). Remarkably, in our study two individuals from Molentargius reached Spain and France, taking advantage of strong southerly and south-easterly winds (Scirocco), which in Sardinia was the second most frequent wind after Mistral. However, the match between seasonal winds and bird migratory destination cannot be extended to the other two colonies. During the migration period, Comacchio experienced mostly north-easterly and easterly winds such as Bora/Grecale and Levante but the majority of birds (62.5%) migrated mostly northeast towards the Lagoon of Venice. Similarly, juvenile flamingos from Margherita di Savoia mostly flew northwards towards the Po Delta and Venice Lagoon (54.5%), not matching the dominant north-westerly wind (Mistral) present at the colony. It therefore appears that other factors, such as the proximity to suitable feeding areas, the presence of large ecological barriers (e.g., seas, deserts and mountains) alongside adult migratory experience in previous years need to be considered when assessing reasons beyond the observed migratory destinations.

### Other weather conditions triggering migration departure and speed

As found in many passerines, waders, ducks, and geese [45], migration departures in flamingos were more frequent around or just after local sunset, with 87% of individuals departing between 8:00 and 10:00 pm (local time). Daytime or late-night departure were rare with only one bird departing at 6:00 pm and three birds departing between 00:00 and 2:00 am. These results confirm behaviours previously observed only in the field. For example, in the Camargue hundreds of flamingos were observed taking off just before sunset to cover long distances during the night [36]. Such observation was also confirmed in African lesser flamingo (*Phoeniconaias minor*), which supports the theory that birds migrate between wetlands only at night [11, 46]. Flying at night is believed to be energetically less costly because of a decrease in air turbulence and a reduction in evaporative water while taking advantage of the cooler and more humid nighttime air, thus reducing the chances of dehydration and overheating [10, 39, 40, 59]. Migration speed was also influenced by humidity with speeds peaking around 70% (Fig. 4). These findings are consistent with other studies which have found that shorebirds flying at altitudes characterized by higher relative humidity are advantaged by a reduction in water loss and fuel stores [29, 71]. Although certain weather variables appeared significant in initiating departure, the time of day remained the most influential predictor. This suggests that flamingos may utilize sunset as a visual cue to take advantage of favorable nighttime conditions (e.g., lower temperatures, higher humidity, lower predation risk, higher propensity to forage [61, 64]) throughout the entire migration journey, rather than departing specifically due to favorable weather conditions coinciding with dusk. Many birds also migrate at night to better rely on celestial cues for navigation during migration [68, 75].

Energetic constraints associated with speed have also been assessed by considering the cumulative migration distance travelled. Higher speeds were achieved at intermediate values of migration distance (range: 103–820 km) suggesting that birds reduced their speed after *ca.* 400 km. While future studies should assess energy expenditure via GPS equipped with accelerometers, we have potential evidence of birds exhausted after flying for long distances with headwinds. Indeed, one individual (ISPR09) migrating from Molentargius to Margherita di Savoia, rested on the water far out at sea for more than 12 h in the Gulf of Naples during strong headwind conditions (25 km/h). A similar observation of flamingos resting at sea has been reported by Amat et al. [2] for an individual resting 89 km east of the coast of Algeria. In Europe there are various reports of exhausted juveniles being found stranded or tired after a long migration, having diverted from the main flock.

### Future directions

Our work investigated the drivers of juvenile post-fledging departure and destination linked to body and weather conditions and ignored the role of adults and individual experience in mixed-age flocks influencing the results observed for juveniles. Systematic field observations of migrating flamingo flocks leaving the breeding site have generally reported a mixture of adults and juveniles suggesting that juveniles may follow older individuals (not necessary their parents; unpublished data reported in [31]) during their first journey, taking advantage of the knowledge and experience of older individuals [5]. A recent work on GPS-tagged Caspian terns *Hydroprogne caspia*, a long distant migrant, demonstrated that juvenile terns stayed close to an adult at all times, most likely to learn the migration route which was faithfully replicated in the following years on their first solo flight as subadults [13]. Some elements of social learning from adult conspecifics are also to be expected in flamingos (as indicated by the selection of stronger tailwinds when crossing long distances) and future studies should assess the influence of adult behaviour on migrating juveniles. We can also expect individual experience (and consequently age) to affect phenology, flight efficiency, and migratory routes (e.g. [24]). Since flamingos are likely to migrate in mixed-age flocks, we can hypothesize that the wind selection we observed for the young individuals in our study is, in fact, applicable to all age groups.

In terms of flight performance, the limit of the 400 km migration threshold after which flight speed declined considerably definitely deserves to be investigated in more detail. Our devices were not equipped with accelerometers, which might have been useful for assessing energy expenditure during long-distance flights, also in relation to sex. Sex-biased differences in migration phenology and speed also suggest the potential existence of sex-biased segregated flocks which could lead to separate migratory destinations. In this context, the large dataset available from ringing activities on flamingos throughout Europe (www.flamingoatlas.org) can certainly be of great help to further investigate several aspects of the migratory behavior of this flag species in association with biologging technologies.

## Conclusion

Our findings support the role of body condition influencing the migration tactics within and among partial migrant bird populations [12, 15]. We provide here the first evidences of potential sexual-differences in post-fledging phenology of flamingos, with males departing earlier than females and of a clear sex-biased differences in migration speeds, with females flying faster. Our analysis confirms the significant role of local tailwinds in triggering migration departure of juvenile flamingos. However, we found less evidence supporting the long-term effects of seasonal winds in determining their displacements towards the non-breeding areas of juvenile flamingos. We argue that the selection of these destinations is primarily influenced by the adults present in mixed flocks, which base their choices on their lifetime experience. For juvenile flamingos, the decision to migrate or stay in the natal area appears to come at great cost, particularly for those whose destinations were over 400 km away, as indicated by the observed depletion in strength of the flight speed beyond this distance. The maintenance of a wetland network in the Mediterranean is therefore crucial for the conservation of this and other waterbird species.

### Supplementary Information

**Additional file 1**. Supplementary information file with background tables and figures. **Table S1.** Sample size used for each analysis according to colony, year and sex. **Table S2.** Summary table describing for each migrating juvenile flamingo (n = 32), date of migration departure and arrival destination, duration of the journey, distance travelled, average and max speed. **Table S3.** Linear model results assessing potential differences in biometrics (tarsus, body mass, wing length) and body condition (Scaled Mass Index) for individuals belonging to different colonies, sex and years. **Figure S1.** Plots of explanatory variables (colony, sex, and year) influencing tarsus length. **Figure S2.** Plots of explanatory variables influencing body mass. **Figure S3.** Plots of explanatory variables mostly influencing wing length. **Figure S4.** Plots of variables influencing Scaled Mass Index according to models shown in Table S2. **Table S4.** Summary results of the logistic regression model assessing effects of body condition (Scaled Mass Index) on the probability of migration. **Table S5.** Linear models assessing correlates of migration phenology for post-fledging flamingos with respect to biometrics, Scaled Mass Index, colony, migration distance, and sex. **Figure S5.** Most important variables predicting migration phenology. **Table S6.** Model summary on the probability of selecting tailwinds or headwinds when leaving the colony in relation to the migratory distance (interaction) accounting for colony, sex, and time of departure. **Table S7.** Model summary of the LMM evaluating sex-related differences in migration speed according to the interaction between sex and tailwind. **Table S8.** Summary of GLMs evaluating the relationship between departure direction of birds and the frequency of wind directions at each colony. **Table S9.** Linear model output evaluating correlates of post-fledging migration distance in greater flamingos. **Figure S6.** Fitted relationship for the most important variables predicting migration distance in post-fledging greater flamingos.

## Data Availability

GPS data are available in the MoveBank repository (project ID: 1,347,882,924) upon request to the corresponding author. The R scripts used for the analyses are also available upon request to the corresponding author.
