# Supplementary material for: Weather, sex and body condition affect post-fledging migration behaviour of the greater flamingo Phoenicopterus roseus
Source: Mov Ecol. 2023 Aug 23;11:51. doi: 10.1186/s40462-023-00409-x (PMC10464070; doi:10.1186/s40462-023-00409-x)
Supplement: Supplementary file 1 — Additional file 1. Supplementary information file with background tables and figures. Table S1. Sample size used for each analysis according to colony, year and sex. Table S2. Summary table describing for each migrating juvenile flamingo (n = 32), date of migration departure and arrival destination, duration of the journey, distance travelled, average and max speed. Table S3. Linear model results assessing potential differences in biometrics (tarsus, body mass, wing length) and body condition (Scaled Mass Index) for individuals belonging to different colonies, sex and years. Figure S1. Plots of explanatory variables (colony, sex, and year) influencing tarsus length. Figure S2. Plots of explanatory variables influencing body mass. Figure S3. Plots of explanatory variables mostly influencing wing length. Figure S4. Plots of variables influencing Scaled Mass Index according to models shown in Table S2. Table S4. Summary results of the logistic regression model assessing effects of body condition (Scaled Mass Index) on the probability of migration. Table S5. Linear models assessing correlates of migration phenology for post-fledging flamingos with respect to biometrics, Scaled Mass Index, colony, migration distance, and sex. Figure S5. Most important variables predicting migration phenology. Table S6. Model summary on the probability of selecting tailwinds or headwinds when leaving the colony in relation to the migratory distance (interaction) accounting for colony, sex, and time of departure. Table S7. Model summary of the LMM evaluating sex-related differences in migration speed according to the interaction between sex and tailwind. Table S8. Summary of GLMs evaluating the relationship between departure direction of birds and the frequency of wind directions at each colony. Table S9. Linear model output evaluating correlates of post-fledging migration distance in greater flamingos. Figure S6. Fitted relationship for the most important variables predicting migration di [file 40462_2023_409_MOESM1_ESM.docx]

**SUPPLEMENTARY MATERIALS**

Weather, sex and body condition affect post-fledging migration behaviour of the greater flamingo *Phoenicopterus roseus*

*Davide Scridel ^1,2,3*^, Simone Pirrello^1^, Simona Imperio^1^, Jacopo G. Cecere^1^, Giuseppe Albanese^4^, Alessandro Andreotti^1^, Giovanni Arveda^5^, Fabrizio Borghesi^6^, Giuseppe La Gioia^7^, Luisanna Massa^8^, Chiara Mengoni^9^, Pierfrancesco Micheloni^1^, Nadia Mucci^9^, Riccardo Nardelli^1^, Sergio Nissardi^10^, Stefano Volponi^11^, Carla Zucca^10^, Lorenzo Serra^1^*

*^1^ Area Avifauna Migratrice (BIO-AVM), Istituto Superiore per la Protezione e la Ricerca Ambientale (ISPRA), via Ca’ Fornacetta 9, 40064 Ozzano dell’Emilia, BO, Italy.*

*^2^ CNR-IRSA National Research Council-Water Research Institute, via del Mulino 19, 20861 Brugherio, MB, Italy.*

*^3^* *Department of Life Sciences, University of Trieste, 341727 Trieste, TS, Italy.*

*^4^ Manfredonia, FG, Italy.*

*^5^* *Comacchio, FE, Italy.*

*^6^ Servizio Tutela Ambiente e Territorio, Ufficio Zone Naturali, Comune di Ravenna, via Berlinguer 30, 48121 Ravenna, RA, Italy.*

*^7^ Associazione Ornitologia Mediterranea, via Saponaro 7, 73100 Lecce, LE, Italy.*

*^8^ Parco Naturale Regionale Molentargius Saline, via La Palma n 9, 09126 Cagliari, CA, Italy.*

*^9^ Area per la Genetica della Conservazione (BIO-CGE), Istituto Superiore per la Protezione e la Ricerca Ambientale (ISPRA), via Ca’ Fornacetta 9, 40064 Ozzano dell’Emilia, BO, Italy.*

*^10^ Anthus s.n.c., via Luigi Canepa 22, 09129 Cagliari, CA, Italy.*

*^11^ Area per i pareri tecnici e per le strategie di conservazione e gestione del patrimonio faunistico nazionale (BIO-CFN), Istituto Superiore per la Protezione e la Ricerca Ambientale (ISPRA), via Ca’ Fornacetta 9, 40064 Ozzano dell’Emilia, BO, Italy.*

**Corresponding author e-mail: dscridel@gmail.com*

**Table S1.** Sample size used for each analysis according to colony, year and sex. Sample size varied from 26 to 42 individuals depending on the analysis considered. Specifics on sample size variation have been noted.

| **Morphological and body condition differences** | | | | | |  |
| --- | --- | --- | --- | --- | --- | --- |
|  | *Year* | *Molentargius* | *Margherita di Savoia* | *Comacchio* | *Number of individuals* | *Specification* |
|  | 2015 | 0F,0M | 0F, 0M | 2F, 2M | 4 | All 42 individuals tagged used for the analysis |
|  | 2016 | 3F,9M | 4F,8M | 2F,9M | 35 |  |
|  | 2017 | 0F,3M | 0F, 0M | 0F, 0M | 3 |  |
|  |  |  |  | Total | 42 |  |
| **Probability of migration** | | | | | |  |
|  | *Year* | *Molentargius* | *Margherita di Savoia* | *Comacchio* | *Number of individuals* | *Specifications* |
|  | 2015 | 0F,0M | 0F, 0M | 2F, 2M | 4 | All migrating (n=32) and non-migrating (n=8) individuals were used in this analysis. Two birds were excluded due to GPS failure. |
|  | 2016 | 3F,9M | 4F,7M | 2F,8M | 33 |  |
|  | 2017 | 0F,3M | 0F, 0M | 0F, 0M | 3 |  |
|  |  |  |  | Total | 40 |  |
| **Migration phenology** | | | | | |  |
|  | *Year* | *Molentargius* | *Margherita di Savoia* | *Comacchio* | *Number of individuals* | *Specifications* |
|  | 2015 | 0F,0M | 0F, 0M | 1F, 2M | 3 | All migratory birds were analyzed (n=32). |
|  | 2016 | 3F,7M | 4F,7M | 2F,3M | 26 |  |
|  | 2017 | 0F,3M | 0F, 0M | 0F, 0M | 3 |  |
|  |  |  |  | Total | 32 |  |
| **Migration distance** | | | | | |  |
|  | *Year* | *Molentargius* | *Margherita di Savoia* | *Comacchio* | *Number of individuals* | *Specifications* |
|  | 2015 | 0F,0M | 0F,0M | 1F, 2M | 3 | 30 migrating birds were analyzed. Two individuals had to be excluded because their GPS transmissions occurred only every 6 hours, which was considered too infrequent to accurately evaluate their migratory distances. |
|  | 2016 | 3F,7M | 4F,7M | 2F, 3M | 26 |  |
|  | 2017 | 0F,1M | 0F,0M | 0F, 0M | 1 |  |
|  |  |  |  | Total | 30 |  |
| **Weather correlates of migratory departure** | | | | | |  |
|  | *Year* | *Molentargius* | *Margherita di Savoia* | *Comacchio* | *Number of individuals* | *Specifications* |
|  | 2015 | 0F,0M | 0F,0M | 1F, 2M | 3 | 30 migrating birds were analyzed. Two individuals had to be excluded because their GPS transmissions occurred only every 6 hours, which was considered too infrequent to accurately assess fine scale weather correlates triggering migration. |
|  | 2016 | 3F,7M | 4F,7M | 2F, 3M | 26 |  |
|  | 2017 | 0F,1M | 0F,0M | 0F, 0M | 1 |  |
|  |  |  |  | Total | 30 |  |
| **Migration speed** | | | | | |  |
|  | *Year* | *Molentargius* | *Margherita di Savoia* | *Comacchio* | *Number of individuals* | *Specifications* |
|  | 2015 | 0F,0M | 0F, 0M | 1F(29), 2M (10) | 3 | This analysis was only performed for consecutive locations in flight. We had to exclude short-distance migratory individuals with only one location in flight. |
|  | 2016 | 3F (12),7M (29) | 4F (22),7M (45) | 1M (1) | 22 |  |
|  | 2017 | 0F,1M (4) | 0F, 0M | 0F, 0M | 1 |  |
|  |  |  |  | Total | 26 |  |
| **Migration direction** | | | | | |  |
|  | *Year* | *Molentargius* | *Margherita di Savoia* | *Comacchio* | *Number of individuals* | *Specifications* |
|  | 2015 | 0F,0M | 0F, 0M | 1F, 2M | 3 | All migratory birds were analyzed (n=32). |
|  | 2016 | 3F,7M | 4F,7M | 2F,3M | 26 |  |
|  | 2017 | 0F,3M | 0F, 0M | 0F, 0M | 3 |  |
|  |  |  |  | Total | 32 |  |

**Table S2.** Summary table describing for each migrating juvenile flamingo (n=32), date of migration departure and arrival destination, duration of the journey, distance travelled, average and max speed (derived only from in-flight locations). For two individuals (ISPR11b, FLAI03b) GPS frequency were much coarser (6 hours) so precise calculation of migration duration and speed were considered too inaccurate to be performed.

| *Flamingo ID* | *Sex* | *Departure from natal colony* | *Stop-over/winter destination* | *Migration duration*  *(hours)* | *Migration distance*  *(km)* | *Average speed*  *(km/h)* | *Max speed*  *(km/h)* |
| --- | --- | --- | --- | --- | --- | --- | --- |
| ISPR07 | Male | Comacchio - 02/09/2016 | Lagoon of Venice - 02/09/2016 | 2 | 103 | NA | NA |
| MALA01 | Male | Comacchio - 06/09/2015 | Corsica - 07/09/2015 | 8 | 358 | 43.4 | 56.0 |
| ISPR04 | Female | Comacchio - 08/09/2016 | Lagoon of Venice - 08/09/2016 | 2 | 136 | NA | NA |
| ISPR06 | Female | Comacchio - 09/09/2016 | Lagoon of Venice - 09/09/2016 | <2 | 124 | NA | NA |
| SPOO01 | Male | Comacchio - 14/09/2016 | Lagoon of Venice - 14/09/2016 | 4 | 103 | 45.0 | 45.0 |
| MALA31 | Male | Comacchio - 19/09/2016 | Lagoon of Venice - 19/09/2016 | 2 | 103 | NA | NA |
| MALA04 | Male | Comacchio - 20/09/2015 | France - 21/09/2015 | 12 | 549 | 45.1 | 61.4 |
| MALA02 | Female | Comacchio - 27/09/2015 | Sardinia - 28/09/2015 | 18 | 813 | 56.1 | 97.2 |
| ISPR12 | Female | M. di Savoia - 08/10/2016 | Po Delta - 09/10/2016 | 8 | 439 | 43.9 | 59.9 |
| ISPR11a | Male | M. di Savoia - 08/10/2016 | Po Delta - 09/10/2016 | 14 | 460 | 28.7 | 42.5 |
| ISPR16 | Female | M. di Savoia - 13/10/2016 | Po Delta - 14/10/2016 | 6 | 437 | 54.6 | 71.3 |
| ISPR15 | Female | M. di Savoia - 16/10/2016 | Tunisia - 17/10/2016 | 14 | 820 | 51.2 | 75.3 |
| MALA14 | Male | M. di Savoia - 23/09/2016 | Sardinia - 24/09/2016 | 14 | 556 | 39.6 | 55.8 |
| MALA06 | Female | M. di Savoia - 23/09/2016 | Po Delta - 24/09/2016 | 8 | 436 | 43.6 | 54.7 |
| ISPR13 | Male | M. di Savoia - 24/09/2016 | Lagoon of Venice - 25/09/2016 | 12 | 572 | 40.9 | 51.4 |
| MALA34 | Male | M. di Savoia - 25/09/2016 | Po Delta - 26/09/2016 | 10 | 447 | 37.2 | 50.0 |
| ISPR17 | Male | M. di Savoia - 28/09/2016 | Tuscany - 29/09/2016 | 8 | 464 | 46.3 | 61.1 |
| ISPR14 | Male | M. di Savoia - 28/09/2016 | Tuscany 29/09/2016 | 10 | 426 | 41.7 | 56.5 |
| ISPR18 | Male | M. di Savoia - 30/09/2016 | Sardinia - 01/10/2016 | 12 | 600 | 42.8 | 54.7 |
| MALA11 | Male | Molentargius - 15/08/2016 | Camargue - 15/08/2016 | 10 | 599 | 49.9 | 56.6 |
| FLAI02 | Male | Molentargius - 17/09/2016 | Tunisia - 18/09/2016 | 4 | 244 | 40.6 | 55.9 |
| ISPR11b | Male | Molentargius - 20/08/2017 | Tunisia - 20/08/2017 | NA | 406 | NA | NA |
| FLAI05 | Male | Molentargius - 21/08/2016 | Tunisia - 21/08/2016 | 4 | 285 | 47.5 | 67.1 |
| MALA13 | Female | Molentargius - 21/08/2016 | Tunisia - 21/08/2016 | 4 | 287 | 49.6 | 68.6 |
| ISPR09 | Female | Molentargius - 23/08/2016 | Apulia - 24/08/2016 | 10 | 467 | 38.9 | 54.8 |
| ISPR08 | Male | Molentargius - 28/08/2016 | Tunisia - 29/08/2016 | 6 | 247 | 56.5 | 60.4 |
| ISPR10 | Male | Molentargius - 29/08/2016 | Tunisia - 30/08/2016 | 8 | 287 | 44.7 | 52.8 |
| SPOO04 | Female | Molentargius - 21/09/2016 | Tunisia - 21/09/2016 | 4 | 288 | 48.0 | 70.9 |
| MALA35 | Male | Molentargius - 23/08/2017 | Tunisia - 24/08/2017 | 8 | 234 | 29.1 | 46.6 |
| FLAI03b | Male | Molentargius - 06/08/2017 | Tunisia - 06/08/2017 | NA | 275 | NA | NA |
| FLAI03a | Male | Molentargius - 17/08/2016 | Tunisia - 18/08/2016 | 4 | 287 | 47.7 | 59.8 |
| FLAI01 | Male | Molentargius - 26/08/2016 | Spain - 27/08/2016 | 16 | 621 | 34.5 | 45.6 |

**Table S3**. Linear model results assessing potential differences in biometrics (tarsus, body mass, wing length) and body condition (Scaled Mass Index) for individuals belonging to different colonies, sex and years. Potential interaction between sex, colony (reference level: Comacchio) and year (reference level: 2015) were preliminary evaluated, but had no influence on the results. Supported variables (i.e. predictors with significant p-values (α = 0.05) and with 95% CI not overlapping zero) are shown in bold.

| **Tarsus** | | | | | | |
| --- | --- | --- | --- | --- | --- | --- |
|  | *Predictors* | *Estimate [95% CI]* | *SE* | *t* | *P* |  |
|  | Intercept | 232.12 [2160.2, 2482.2] | 79.38 | 29.24 | <0.0001 |  |
|  | **Sex (male)** | **15.25 [42.48, 262.67]** | **5.42** | **2.81** | **0.008** |  |
|  | **Colony (Margherita di Savoia)** | **-98.80 [-1115.49, -860.69]** | **6.28** | **-15.73** | **<0.0001** |  |
|  | **Colony (Molentargius)** | **-72.91 [-855.66, -602.61]** | **6.23** | **-11.68** | **<0.0001** |  |
|  | **Year (2016)** | **86.85 [688.39, 1048.60]** | **8.88** | **9.78** | **<0.0001** |  |
|  | **Year (2017)** | **92.53 [654.67, 1196.03]** | **13.47** | **6.93** | **<0.0001** |  |
| **Body mass** | | | | | | |
|  | *Predictors* | *Estimate [95% CI]* | *SE* | *t* | *P* |  |
|  | Intercept | 2216.22 [1846.77, 2585.67] | 182.17 | 12.17 | <0.0001 |  |
|  | Sex (male) | 67.55 [-185.10, 320.21] | 124.58 | 0.54 | 0.591 |  |
|  | Colony (Margherita di Savoia) | -276.13 [-568.49, 16.23] | 144.16 | -1.92 | 0.063 |  |
|  | **Colony (Molentargius)** | **-435.92 [-726.28, -145.57]** | **143.17** | **-3.05** | **0.004** |  |
|  | **Year (2016)** | **914.87 [501.55, 1328.18]** | **203.8** | **4.49** | **<0.0001** |  |
|  | **Year (2017)** | **985.48 [364.29, 1606.67]** | **306.29** | **3.22** | **0.003** |  |
| **Wing length** | | | | | | |
|  | *Predictors* | *Estimate [95% CI]* | *SE* | *t* | *P* |  |
|  | Intercept | 331.96[307.30, 356.62] | 12.15 | 12.17 | <0.0001 |  |
|  | Sex (male) | 5.56 [-11.29, 22.42] | 8.31 | 0.54 | 0.051 |  |
|  | **Colony (Margherita di Savoia)** | **-67.42 [-86.94, -47.91]** | **9.62** | **-1.92** | **<0.0001** |  |
|  | Colony (Molentargius) | 13.44 [-59.38, 32.81] | 9.55 | -3.05 | 0.168 |  |
|  | **Year (2016)** | **34.25 [6.66, 61.83]** | **13.6** | **4.49** | **0.016** |  |
|  | Year (2017) | 41.02 [-0.43, 82.48] | 20.44 | 3.22 | 0.052 |  |
| **Scaled Mass Index** | | | | | | |
|  | *Predictors* | *Estimate [95% CI]* | *SE* | *t* | *P* |  |
|  | Intercept | 2348.23 [2062.54, 2633.93] | 140.87 | 16.67 | <0.0001 |  |
|  | Sex (male) | 180.34 [-15.03, 375.72] | 96.33 | 1.87 | 0.061 |  |
|  | **Colony (Margherita di Savoia)** | **703.37 [477.29, 929.45]** | **111.47** | **6.31** | **<0.0001** |  |
|  | Colony (Molentargius) | 219.59 [-4.94, 444.12] | 110.71 | 1.98 | 0.055 |  |
|  | Year (2016) | 140.66 [-178.94, 460.28] | 157.59 | 0.89 | 0.378 |  |
|  | Year (2017) | 161.12 [-319.23, 641.47] | 236.85 | 0.68 | 0.5 |  |

**
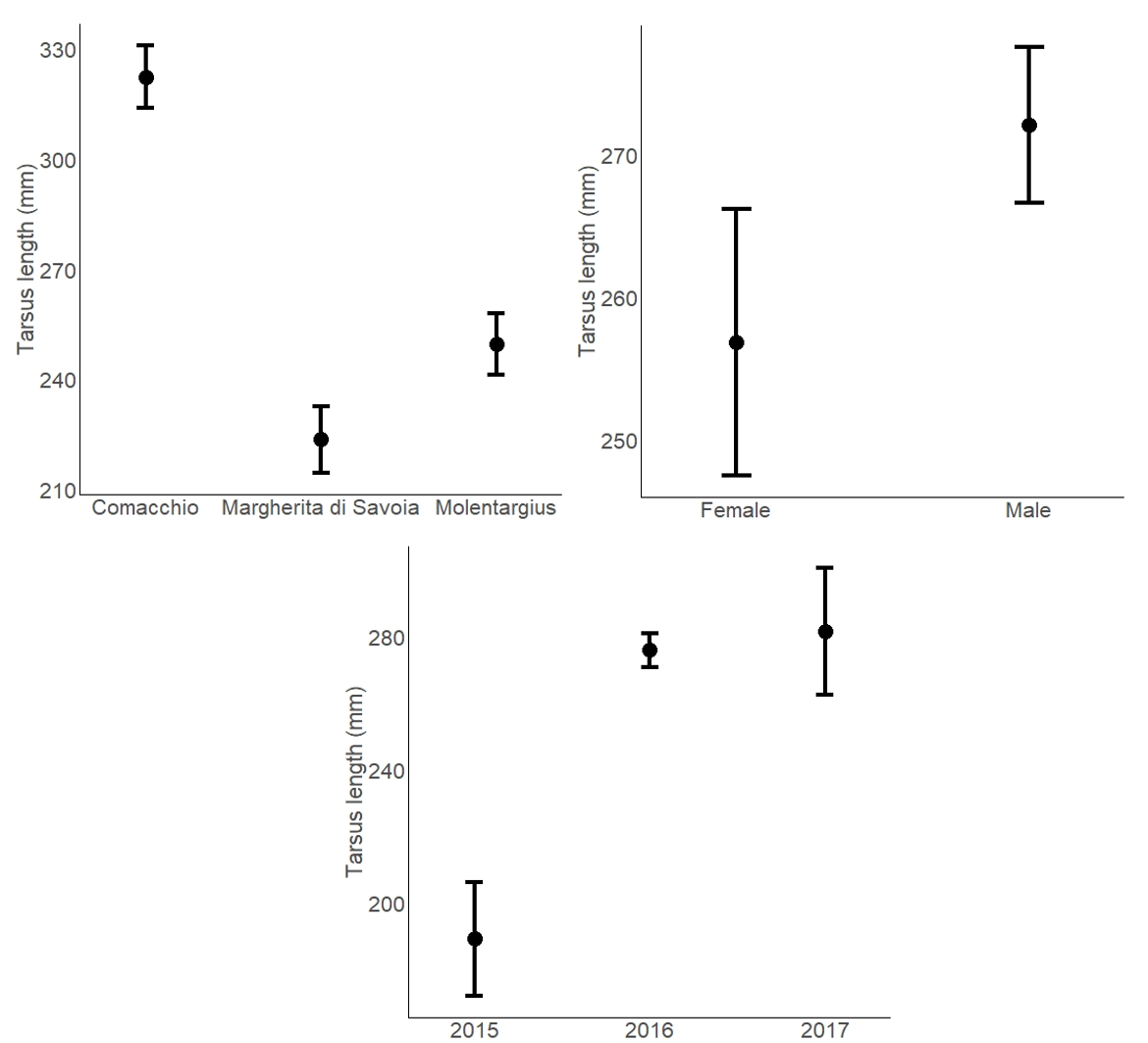
**

**Figure S1**. Plots of explanatory variables (colony, sex, and year) influencing tarsus length according to models shown in Table S2.


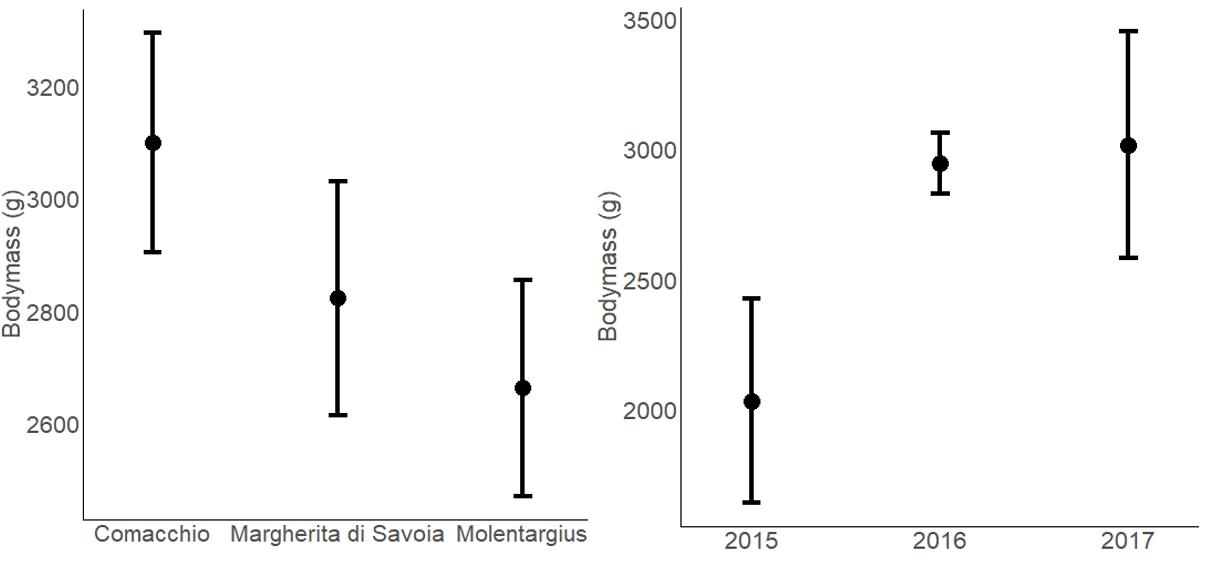


**Figure S2**. Plots of explanatory variables (colony and year) influencing body mass according to models shown in Table S2.


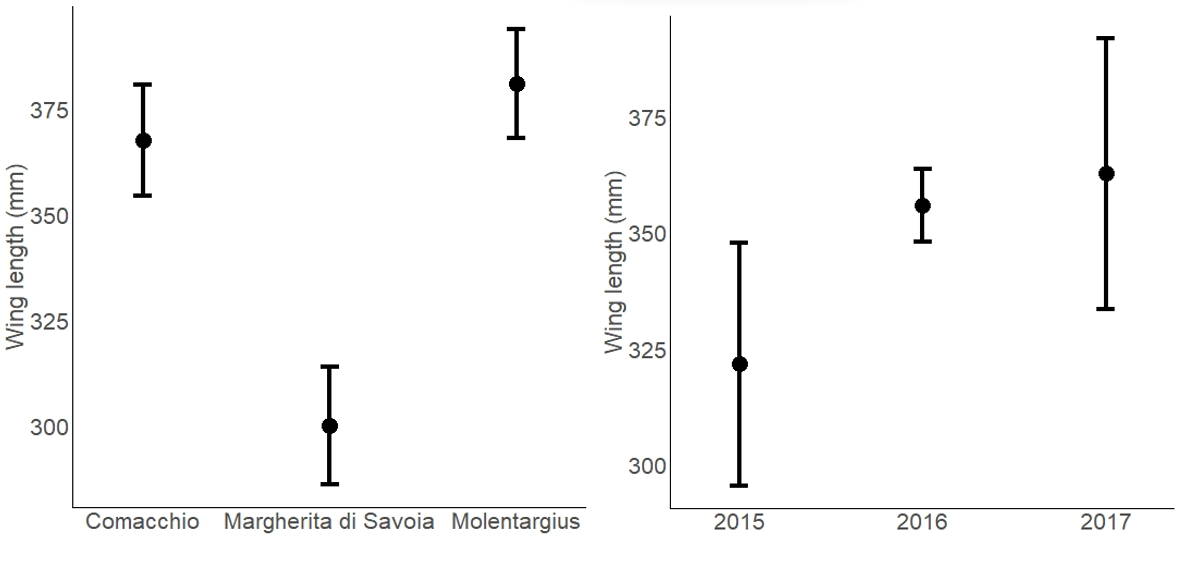
**Figure S3**. Plots of explanatory variables (colony and year) influencing wing length according to models shown in Table S2.

**
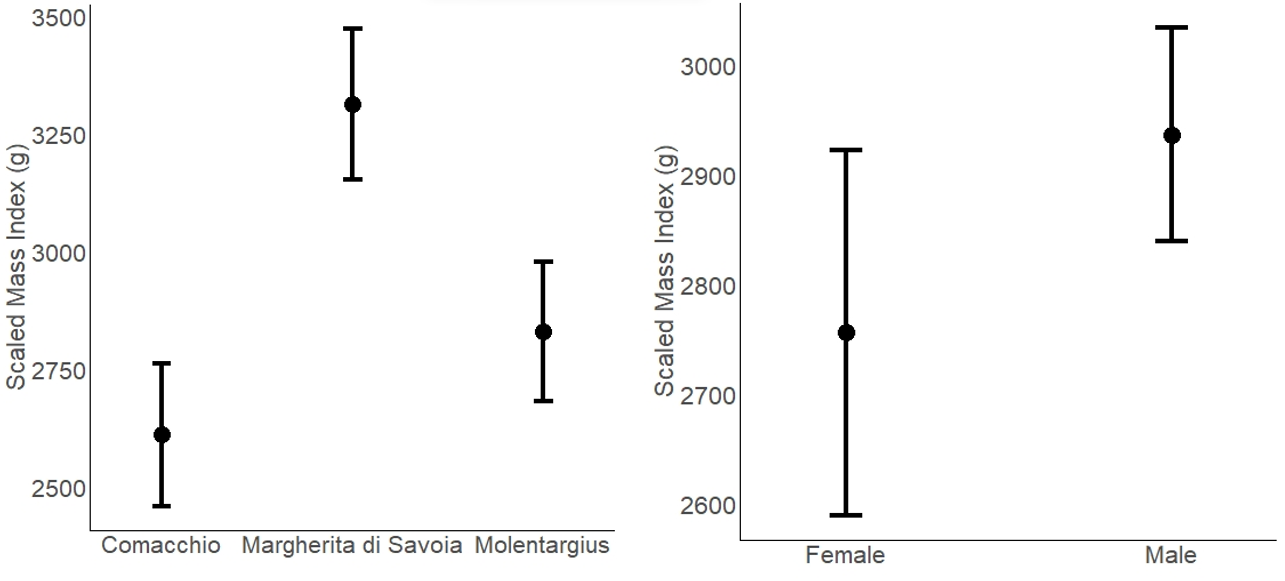
**

**Figure S4**. Plots of variables (colony and sex) influencing Scaled Mass Index according to models shown in Table S2.

**Table S4.** Summary results of the logistic regression model assessing effects of body condition (Scaled Mass Index) on the probability of migration. The response variable represented a dichotomous response coded as “0” for juveniles that did not migrate (n=8) and “1” for migrating ones (n=32). Standardized estimates are shown alongside 95% confidence intervals, standard error (SE), test statistics (Chi-squared test), degrees of freedom (df) and p-value (*P*).

| **Migration probability** | | | | | |  |
| --- | --- | --- | --- | --- | --- | --- |
|  | *Predictors* | *Estimate [95% CI]* | *SE* | *X^2^* | *df* | *P* |
|  | Intercept | -10.76 [-33.58, -4.34] | 4.52 | - | - | 0.034 |
|  | **Scaled Mass Index** | **0.004 [0.001, 0.008]** | **0.001** | **10.26** | **1** | **0.001** |

**Table S5.** Linear models assessing correlates of migration phenology for post-fledging flamingos with respect to biometrics, Scaled Mass Index, colony, migration distance, and sex (explanatory variables). The response variable was the date of departure from the colony expressed as day of the year since 1^st^ of January. Standardized estimates are shown alongside 95% confidence intervals, standard error (SE), test statistics (t-distributed) and p-value (*P*). Supported variables (i.e. predictors with significant p-values (α = 0.05) and with 95% CI not overlapping zero) are shown in bold. Effects of potential interaction between biometrics, colony and sex were preliminary assessed and excluded as negligible.

| **Tarsus** | | | | | |
| --- | --- | --- | --- | --- | --- |
|  | *Predictors* | *Estimate [95% CI]* | *SE* | *t* | *P* |
|  | Intercept | 239.6 [201.38, 277.83] | 18.52 | 12.94 | <0.0001 |
|  | Colony (Comacchio) | 17.62 [-0.19, 35.44] | 8.63 | 2.04 | 0.052 |
|  | **Colony (Margherita di Savoia)** | **33.98 [24.05, 43.91]** | **4.81** | **7.07** | **<0.0001** |
|  | Sex (male) | -4.35 [-13.69, 4.98] | 4.53 | -0.96 | 0.345 |
|  | Tarsus | -0.11 [-0.39, 0.15] | 0.13 | -0.90 | 0.375 |
|  | Migration distance | 0.43 [-5.52, 6.40] | 2.89 | 0.15 | 0.88 |
|  | Year (2016) | 5.1 [-29.04, 39.25] | 16.55 | 0.31 | 0.76 |
|  | Year (2017) | -5.82 [-43.69, 32.03] | 18.34 | -0.32 | 0.753 |
| **Body mass** | | | | | |
|  | *Predictors* | *Estimate [95% CI]* | *SE* | *t* | *P* |
|  | Intercept | 258.04 [231.57, 284.51] | 12.82 | 20.12 | <0.0001 |
|  | Colony (Comacchio) | 10.61 [-4.21, 25.43] | 7.18 | 1.48 | 0.153 |
|  | **Colony (Margherita di Savoia)** | **33.96 [23.98, 43.94]** | **4.84** | **7.02** | **<0.0001** |
|  | Sex (male) | -6.99 [-15.18, 1.19] | 3.97 | -1.76 | 0.091 |
|  | Bodymass | 1.70 [-3.17, 6.58] | 2.36 | 0.72 | 0.476 |
|  | Migration distance | 0.02 [-5.89, 5.92] | 2.86 | 0.01 | 0.995 |
|  | Year (2016) | -11.27 [-35.51, 12.95] | 11.74 | -0.96 | 0.346 |
|  | Year (2017) | -22.36 [-50.49,5.78] | 13.63 | -1.64 | 0.114 |
| **Wing length** | | | | | |
|  | *Predictors* | *Estimate [95% CI]* | *SE* | *t* | *P* |
|  | Intercept | 258.74 [190.09, 327.37] | 33.256 | 7.78 | <0.0001 |
|  | Colony (Comacchio) | 12.55 [-1.29, 26.40] | 6.710 | 1.87 | 0.073 |
|  | **Colony (Margherita di Savoia)** | **32.33 [10.35, 54.29]** | **10.64** | **3.04** | **0.005** |
|  | Sex (male) | -6.24 [-14.77, 2.29] | 4.135 | -1.51 | 0.144 |
|  | Wing length | -0.02 [-0.22, 0.19] | 0.100 | -0.18 | 0.861 |
|  | Migration distance | 0.13 [-6.06, 6.32] | 2.999 | 0.04 | 0.966 |
|  | Year (2016) | -5.63 [-30.81, 19.55] | 12.203 | -0.46 | 0.649 |
|  | Year (2017) | -16.92 [-46.41, 12.58] | 14.292 | -1.18 | 0.248 |
| **Scaled Mass Index** | | | | | |
|  | *Predictors* | *Estimate [95% CI]* | *SE* | *t* | *P* |
|  | Intercept | 256.95 [234.92, 278.98] | 10.67 | 24.07 | <0.0001 |
|  | **Colony (Comacchio)** | **15.91 [2.19, 29.61]** | **6.64** | **2.39** | **0.025** |
|  | **Colony (Margherita di Savoia)** | **29.43 [18.37, 40.50]** | **5.36** | **5.49** | **<0.0001** |
|  | **Sex (male)** | **-8.95 [-17.23, -0.68]** | **4.01** | **-2.23** | **0.035** |
|  | Scaled Mass Index | 4.43 [-1, 9.88] | 0.01 | 1.68 | 0.105 |
|  | Migration distance | 0.33 [-5.33, 5.99] | 2.75 | 0.12 | 0.904 |
|  | Year (2016) | -8.41 [-28.26, 11.44] | 9.62 | -0.87 | 0.39 |
|  | Year (2017) | -18.72 [-43.08, 5.63] | 11.80 | -1.59 | 0.125 |


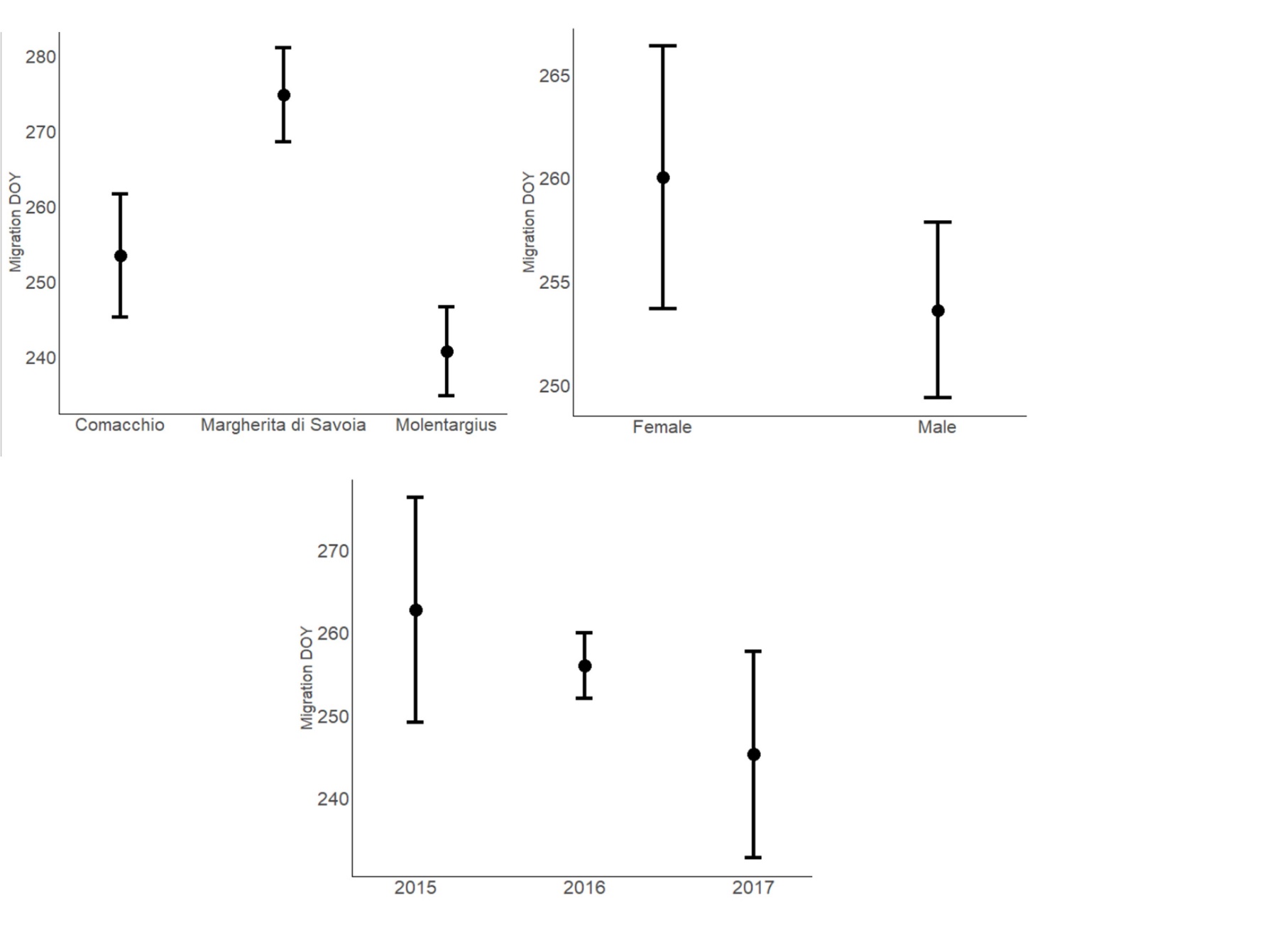


**Figure S5.** Most important variables (colony, sex, and year) predicting migration phenology (departure date, expressed as day of the year, 1 = 1st of January; “Migration DOY”).

**Table S6.** Model summary on the probability of selecting tailwinds or headwinds when leaving the colony in relation to the migratory distance (interaction) accounting for colony, sex, and time of departure.

| **Tailwind and migration distance interaction** | | | | | | |
| --- | --- | --- | --- | --- | --- | --- |
|  | *Predictors* | *Estimate [95% CI]* | *SE* | *X* | *df* | *P* |
|  | Intercept | -5.29 | 0.85 | *38.55* | 1 | <0.0001 |
|  | **Tailwind** | **1.66 [0.98, 2.33]** | **0.34** | **23.44** | **1** | **<0.0001** |
|  | **Migratory distance** | **-1.03 [-1.79, -0.27]** | **0.3** | **7.07** | **1** | **0.007** |
|  | **Colony (Margherita di Savoia)** | **2.02 [0.59, 3.46]** | **0.73** | **8.54** | **2** | **0.005** |
|  | Colony (Molentargius) | 0.51 [-0.59, 1.62] | 0.56 | 8.54 | 2 | 0.365 |
|  | Sex | 0.58 [-0.38, 1.54] | 0.49 | 1.4 | 1 | 0.23 |
|  | **Hours before sunset** | **4.08 [203, 6.13]** | **1.04** | **15.32** | **1** | **<0.0001** |
|  | **Hours before sunset^2^** | **-2.67 [-4.09, -1.25]** | **0.72** | **13.68** | **1** | **<0.0001** |
|  | **Tailwind*Migratory distance** | **0.84 [0.20, 1.48]** | **0.3** | **6.66** | **1** | **0.009** |

**Table S7** Model summary of the LMM evaluating sex-related differences in migration speed according to the interaction between sex and tailwind.

| **Migration speed** | |  |  |  |  |  |
| --- | --- | --- | --- | --- | --- | --- |
|  | *Predictors* | *Estimate [95% CI]* | *SE* | *F* | *df* | *P* |
|  | Intercept | 45.62 [39.93, 51.32] | *2.9* | *185.18* | *1* | <0.0001 |
|  | **Tailwind** | **0.89 [0.56, 1.22]** | **0.17** | **26.58** | **1** | **<0.0001** |
|  | Sex | -2.75 [-9.63, 4.14] | 3.51 | 0.49 | 1 | 0.45 |
|  | **Sex*Tailwind** | **-0.70 [-1.21, -0.19]** | **0.26** | **7.08** | **1** | **0.009** |

**Table S8** Summary of GLMs evaluating the relationship between departure direction of birds and the frequency of wind directions (both categorised into 6 classes, each spanning 60°) at each colony.

| **Comacchio** | | | | | | |
| --- | --- | --- | --- | --- | --- | --- |
|  | *Predictors* | *Estimate [95% CI]* | *SE* | *X^2^* | *df* | *P* |
|  | Intercept | -0.5 [-2.10, 1.61] | 0.92 | 0.003 | 1 | 0.956 |
|  | Frequency of winds | 0.001 [-0.006, 0.008] | 0..003 | 0.17 | 1 | 0.683 |
| **Molentargius** | | | | | | |
|  | *Predictors* | *Estimate [95% CI]* | *SE* | *X^2^* | *df* | *P* |
|  | Intercept | -2.14 [-4.55, -0.51] | 0.99 | 4.64 | 1 | 0.031 |
|  | **Frequency of winds** | **0.004 [0.002, 0.006]** | **0.0009** | **15.8** | **1** | **<0.0001** |
| **Margherita di Savoia** | | | | | | |
|  | *Predictors* | *Estimate [95% CI]* | *SE* | *X^2^* | *df* | *P* |
|  | Intercept | 2.65 [1.40, 3.83] | 0.62 | 18.2 | 1 | <0.0001 |
|  | **Frequency of winds** | **-0.005 [-0.009, -0.002]** | **0.002** | **7.08** | **1** | **0.008** |

**Table S9** Linear model output evaluating correlates of post-fledging migration distance in greater flamingos. Response variable was the cumulative distance performed by each flamingo associated to the mean environmental conditions while accounting for sex and colony (explanatory variables). Standardized estimates are shown alongside 95% confidence intervals, test statistics (t-distributed) and p-value (*P*). Predictors with significant p-values (α = 0.05) and with 95% confidence intervals non-overlapping zero are shown in bold.

| **Migration distance** | | | | | |
| --- | --- | --- | --- | --- | --- |
|  | *Predictors* | *Estimate [95% CI]* | *SE* | *t* | *P* |
|  | Intercept | 617.28 [358.49, 876.07] | 121.42 | 5.084 | <0.0001 |
|  | Rain | 68.87 [-29.41, 167.16] | 46.11 | 1.494 | 0.156 |
|  | Humidity | 54.97 [-43.07, 153.02] | 46.00 | 1.195 | 0.251 |
|  | Temperature | 16.91 [-170.22, 204.05] | 87.80 | 0.193 | 0.85 |
|  | Crosswind | -16.54 [-131.09, 98.01] | 53.74 | -0.308 | 0.762 |
|  | Surface air pressure | 33.89 [-43.92, 111.70] | 36.51 | 0.928 | 0.368 |
|  | Tailwind | -43.92 [-98.71, 90.87] | 44.47 | -0.088 | 0.931 |
|  | Cloud cover (high) | -25.13 [-134.33, 84.07] | 51.23 | -0.491 | 0.631 |
|  | Cloud cover (medium) | -48.75 [-159.1, 61.59] | 51.77 | -0.942 | 0.361 |
|  | Day of migration | -7.56 [-143.38, 128.26] | 63.72 | -0.119 | 0.907 |
|  | **Colony (Margherita di Savoia)** | **351.86 [90.25, 613.47]** | **122.74** | **2.867** | **0.012** |
|  | Colony (Molentargius) | 195.62 [-86.96, 477.94] | 132.45 | 1.477 | 0.160 |
|  | **Year (2016)** | **-428.34 [786.31, -70.13]** | **167.95** | **-2.55** | **0.022** |
|  | **Year (2017)** | **-571.93 [-1070.75, -73.13]** | **234.02** | **-2.444** | **0.027** |
|  | Sex (Male) | -54.53 [-203.05, 93.98] | 69.68 | -0.783 | 0.446 |


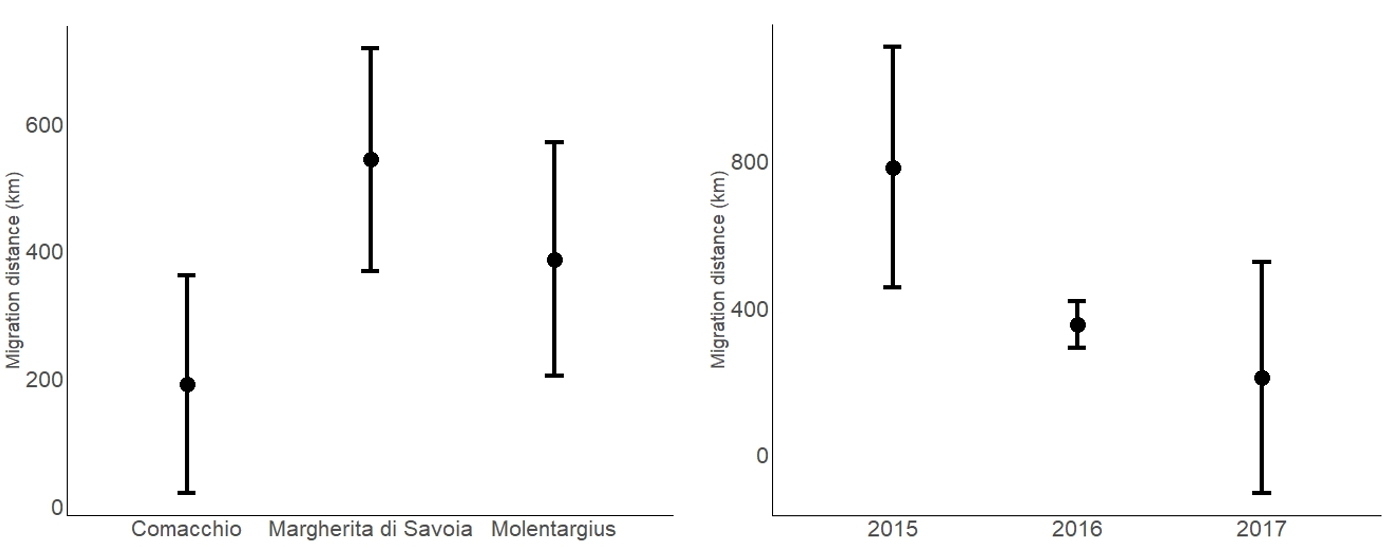


**Figure S6.** Fitted relationship for the most important variables (colony and year) predicting migration distance in post-fledging greater flamingos.
